# Carboxylic acid stimulated silver shell isomerism in a triple core–shell Ag_84_ nanocluster†
†Electronic supplementary information (ESI) available: IR, ^13^C NMR, UV-Vis, EDS and PXRD data, and details of the data collection and structure refinements, and crystal data. CCDC 1878911 and 1878912 for **SD/Ag84a** and **SD/Ag84b**. For ESI and crystallographic data in CIF or other electronic format see DOI: 10.1039/c8sc05666h


**DOI:** 10.1039/c8sc05666h

**Published:** 2019-03-29

**Authors:** Zhi Wang, Hao-Tian Sun, Mohamedally Kurmoo, Qing-Yun Liu, Gui-Lin Zhuang, Quan-Qin Zhao, Xing-Po Wang, Chen-Ho Tung, Di Sun

**Affiliations:** a Key Laboratory of Colloid and Interface Chemistry , Ministry of Education , School of Chemistry and Chemical Engineering , State Key Laboratory of Crystal Materials , Shandong University , Jinan , 250100 , People's Republic of China . Email: dsun@sdu.edu.cn; b College of Chemical Engineering and Materials Science , Zhejiang University of Technology , Hangzhou , 310032 , People's Republic of China . Email: glzhuang@zjut.edu.cn; c Institut de Chimie de Strasbourg , Université de Strasbourg , CNRS-UMR 7177 , 4 rue Blaise Pascal , 67008 Strasbourg Cedex , France; d College of Chemical and Environmental Engineering , Shandong University of Science and Technology , Qingdao , 266590 , People's Republic of China

## Abstract

Isomerization is highly important in all aspects of science, yet it is rarely observed in nanoscience. Here, we synthesized a unique triple core–shell Ag_84_ nanocluster displaying isomerism, which is controlled by different carboxylic acids and a one-way transformation (**SD/Ag84a** → **SD/Ag84b**). The innermost core is a rare Ag_10_ nanocluster which comprises an Ag_6_ octahedral unit as seen in face-centred cubic (fcc) silver metal and four capped Ag atoms. It templates two crescent-shaped polyoxometalate (W_7_O_26_)^10–^ shells which are then enclosed in a shell of silver shaped as rugby balls. The organic ligands (^i^PrS^–^, ^*n*^PrCOO^–^ and PhCOO^–^) finally shield the metallic clusters. Due to slight differences in structure at two poles and the steric hindrance of ^*n*^PrCOO^–^ and PhCOO^–^, **SD/Ag84a** and **SD/Ag84b** adopt the shapes of flat-headed and cuspidal prolate spheres, respectively. Interestingly, PhCOOH is dominant over ^*n*^PrCOOH whereby crystals of **SD/Ag84b** were isolated if PhCOOH is added during the synthesis of **SD/Ag84a**. This demonstrates that PhCOOH not only alters the organic coats but also induces metal shell re-organization. This work reveals carboxylate-controlled skeletal isomerism in silver nanoclusters for the first time, thus deepening the understanding of silver nanocluster assembly, flexibility and reactivity.

## Abstract 2

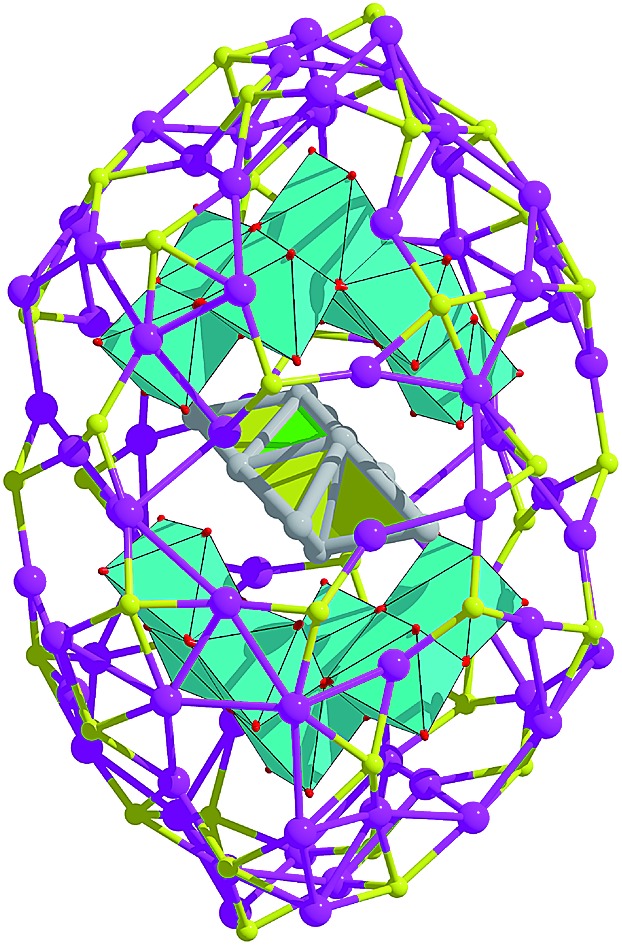
A unique triple core–shell Ag_84_ nanocluster displaying isomerism, which is controlled by different carboxylic acids and a one-way transformation (**SD/Ag84a** and **SD/Ag84b**).

## Introduction

Isomerization plays a vital role in many fundamental biological processes in nature such as ligand binding, enzymatic catalysis, protein folding and photosynthesis.^1^ Diverse isomerizations in inorganic, organic, and supramolecular chemistry have also attracted attention since isomers—compounds with the same atoms arranged in different manners—may deliver substantially different physicochemical properties and chemical reactivities without changing their compositions;^2^ for example, the sweetness of optically active sugars. For polyatomic systems, such as metal nanoclusters, isomerization is not a highly probable event because (i) structural variation usually involves the re-organization of several dozens of metal atoms and motion of several ligands and (ii) unravelling atomic-precision in nanometric structures or even macromolecules is still a major challenge. Thus, it is difficult to experimentally achieve atomic-level isomerization of specific sites/regions in a cluster without destroying the other parts of the main skeleton. An important breakthrough was made in 2015 by Jin and Wu who reported the first genuine gold nanocluster isomers, Au_38T_ and Au_38Q_.^3^ Following this work, their group reported ligand-induced core isomerization in two thiolate-protected Au_28_ nanoclusters.^4^ Tsukuda also discovered anion-packing induced isomerization between crown- and butterfly-Au_9_.^5^ Although there has been sporadic advances in Au nanocluster isomerization recently,^6^ those for silver are yet to be achieved.

The development of silver nanoclusters has expedited the establishment of general synthetic methodologies such as anion-templation and geometric polyhedral principles.^7^ The gradual accumulation of knowledge about silver nanocluster synthesis is also a reminder for us to revisit the ligand strategy^8^ based on the classic hard-soft-acid-base (HSAB) theory,^9^ that is controlling the ‘hardness’ of carboxylic acids in competition with ‘soft’ thiolates, allowing flexibility of the overall silver nanoclusters. The abundant availability of commercial alkyl or aryl carboxylates provides ample options to tune the structure and flexibility of silver nanoclusters through steric hindrance or/and electronic effects. Flexible silver nanoclusters may produce isomers under specific stimuli including acid, base, ligand exchange and so on. Given this situation, using this soft/hard double-ligand strategy in the rational synthesis of isomeric silver nanoclusters is very attractive and challenging.

This study is born out of the successes of the recent afore-mentioned synthetic strategy achieving two Ag_84_ nanoclusters (**SD/Ag84a** and **SD/Ag84b**). Their impressive structures comprise a Ag_10_ nanocluster core, a pair of novel crescent-shaped (W_7_O_26_)^10–^ shells and a 74-silver outer shell, thus establishing a novel common rugby-ball shaped three-shell [Ag_10_@(W_7_O_26_)_2_@Ag_74_] motif that differs in skeletal organization and ligand coverage at the two poles. Their flat-headed and cuspidal prolate spherical structures, respectively, are likely driven by the different steric hindrances between ^*n*^PrCOO^–^ and PhCOO^–^. Although the organic shells are different, these two Ag_84_ nanoclusters have identical elemental contents, and hence, they belong to pseudo-isomers. What’s more interesting is that we can isolate **SD/Ag84b** in the mother solution of **SD/Ag84a** by adding PhCOOH at the second-step reaction, which demonstrates that PhCOOH not only changes the organic coverage but also induces metal shell distortion or re-organization.

## Results and discussion

### Synthesis of **SD/Ag84a** and **SD/Ag84b**

**Scheme 1:**
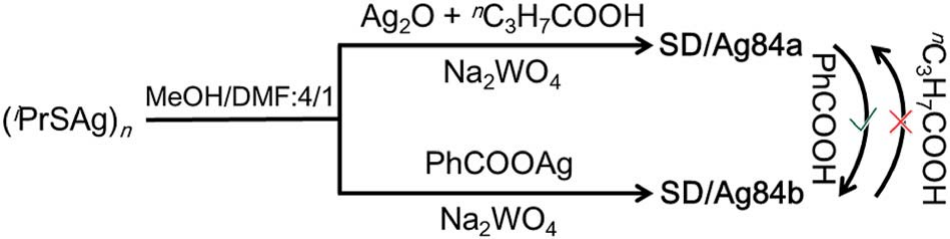
Schematic representation of the assembly and conversion of **SD/Ag84a** and **SD/Ag84b**.

The details of the synthesis of **SD/Ag84a** and **SD/Ag84b** are given in the ESI.† Briefly, (^i^PrSAg)_*n*_, silver salts, RCOOH, and Na_2_WO_4_ were mixed in MeOH/DMF (v/v = 4/1) and reacted under solvothermal conditions (Scheme 1). Red crystals were collected and characterized by single-crystal and powder X-ray diffraction (SCXRD, PXRD), IR, UV-Vis, elemental analyses, and luminescence.

### X-ray structures of **SD/Ag84a** and **SD/Ag84b**

**Fig. 1:**
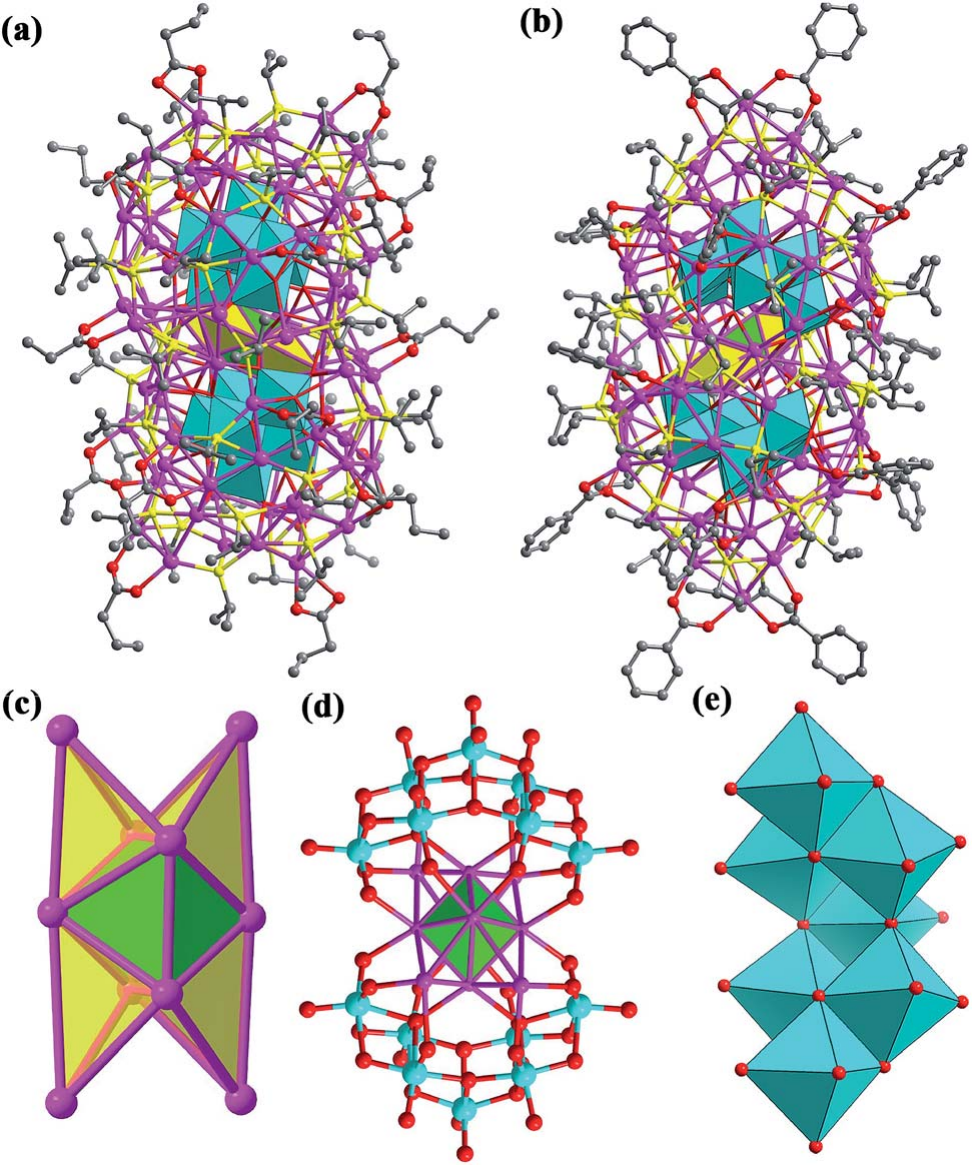
Structures of the clusters **SD/Ag84a** (a) and **SD/Ag84b** (b). (c) The polyhedral mode showing an Ag_6_ octahedron (green) capped by four additional silver tetrahedra (yellow) to form the Ag_10_ kernel; (d) two (W_7_O_26_)^10–^ anions wrapping an Ag_10_ kernel and (e) polyhedral mode showing the structure of a (W_7_O_26_)^10–^ anion. Color labels: purple, Ag; cyan, W; yellow, S; gray, C; and red, O.

Both **SD/Ag84a** and **SD/Ag84b** crystallize in the triclinic *P*1[combining macron] space group with half of the cluster existing as the asymmetric unit. Apart from the carboxylate group they have similar formulae of [Ag_10_@(W_7_O_26_)_2_@Ag_74_S_2_(^i^PrS)_40_(^*n*^PrCOO)_18_]·2CH_3_OH (**SD/Ag84a**) and [Ag_10_@(W_7_O_26_)_2_@Ag_74_S_2_(^i^PrS)_40_(PhCOO)_18_] (**SD/Ag84b**). Their overall appearance is a prolate sphere with similar dimensions of about 1.1 × 2.5 nm. Their coverage by organic ligands is also similar to 40 ^i^PrS^–^ and 18 RCOO^–^ ligands (Fig. 1a and b). Both Ag_84_ clusters consist of an Ag_74_ outer shell and two crescent-shaped (W_7_O_26_)^10–^ inner shells clamping a subnanometer Ag_10_ kernel. The Ag_10_ kernel is constructed from an octahedral Ag_6_ core by adding four Ag caps at the triangular faces (Fig. 1c). Two (W_7_O_26_)^10–^ anions symmetrically wrap the Ag_10_ kernel through Ag–O bonds (Fig. 1d). Each (W_7_O_26_)^10–^ anion is formed by seven edge-sharing WO_6_ octahedra (Fig. 1e) and coordinates to 32 silver atoms in total (Fig. S1†). Such (W_7_O_26_)^10–^ anions are not only observed in the POM@Ag cluster family for the first time but also have never been documented in classical POM chemistry. Two *in situ* generated μ_6_-S^2–^ ions were inserted into the equatorial waist section, consolidating the linkage between the inner Ag_10_ kernel and the Ag_74_ outer shell (Fig. S2†). We listed several important geometry parameters of **SD/Ag84a** and **SD/Ag84b** such as Ag···Ag distances in the Ag_10_ kernel and the Ag_74_ shell, Ag–S_ligand_, Ag–S_sulfide_, Ag–O_carboxylate_, Ag–O_POM_, and W–O bond lengths, and coordination modes of ligands and (W_7_O_26_)^10–^ for comparison (Table S1†).

Notably, such an Ag_10_ kernel is observed for the first time, although its innermost subvalent Ag_6_^4+^ octahedron has been reported in silver nanoclusters^10^ and some inorganic compounds such as Ag_6_Ge_10_P_12_, Ag_5_GeO_4_, Ag_5_SiO_4_ and Ag_6_O_2_.^11^ The formation of such subvalent Ag nanoclusters is related to the reductive effect of DMF, which has been recognized as a key factor in the controlled synthesis of multiple-twin silver nanocrystals by reducing Ag^+^ → Ag^0^.^12^ Based on the digested ^13^C NMR spectra of reaction mother solutions of **SD/Ag84a** and **SD/Ag84b** (Fig. S3 and S4†), we observed typical C_carboxyl_ resonances at *δ* = 163.16 and 163.04 ppm corresponding to the oxidization product of DMF, Me_2_NCOOH.^13^ In the same region, the expected C_aldehyde_ of DMF and C_carboxyl_ of ^*n*^PrCOOH and PhCOOH were also observed at *δ* = 164.39, 176.47, and 168.95 ppm, respectively. These results provided experimental evidence that the redox reaction happened in such a complicated self-assembly process. As the smallest unit in fcc bulk silver metal, the subnanometer Ag_6_ octahedron with eight exposed [111] facets can be seen as an embryonic state of any other bigger silver nanocrystals and thus is of particular interest in the field of nanoparticles.^14^ The subnanometer Ag_10_ kernel trapped in **SD/Ag84a** and **SD/Ag84b** can be seen as the Ag_6_ “nuclei” grown by adding four tetrahedra on its four [111] facets, manifesting the atomic-level silver nanocrystal growth route, that is, stepwise growth of tetrahedral caps on specific facets. Such a growth route is quite similar to that proposed for larger decahedral and icosahedral silver nanocrystals by the Tsuji group.^15^ This result thus sheds light on the atomic details of the growth of silver nanocrystals in the embryonic stage.

**Fig. 2:**
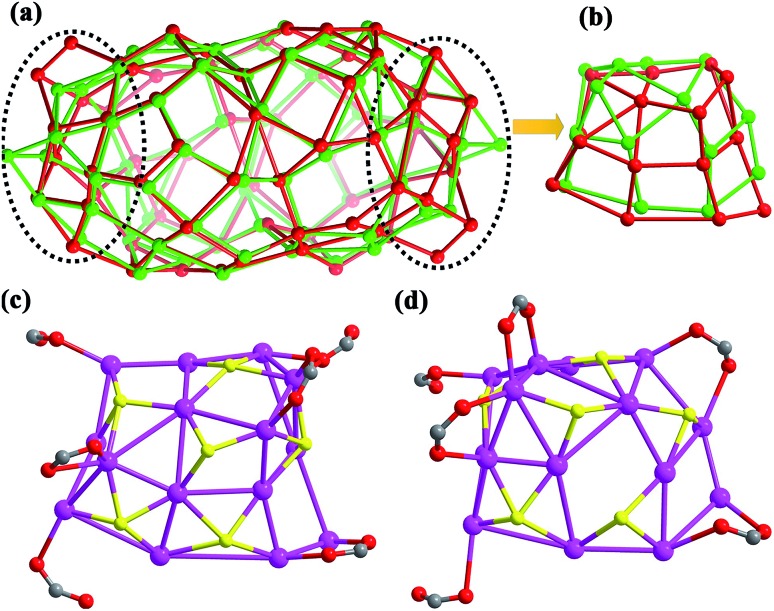
(a) The superposed Ag_74_ shells of **SD/Ag84a** (red) and **SD/Ag84b** (green). (b) The top views of silver polygons at the pole regions of **SD/Ag84a** (red) and **SD/Ag84b** (green). The top views of ligand distributions at the pole region of **SD/Ag84a** (c) and **SD/Ag84b** (d).

Upon further carefully checking and comparing the structural features such as ligand distributions and skeletons of these two silver nanoclusters, we surprisingly found that the distinct differences of silver skeletons between **SD/Ag84a** and **SD/Ag84b** are in the polar sections and have nothing to do with the inner Ag_10_ kernel and (W_7_O_26_)^10–^, although the silver polygons on the other regions of surfaces are almost identical with slight dislocations and distortions. The Ag_74_ shells in **SD/Ag84a** and **SD/Ag84b** can be described as flat-headed (red skeleton in Fig. 2a) and cuspidal (green skeleton in Fig. 2a) prolate spheres, respectively. The superposed Ag_74_ shells, especially in the polar regions (Ag_15_ caps, Fig. 2b), showed silver polygon migrations and severe distortions, which are caused by the different distributions of ^i^PrS^–^ and RCOO^–^ ligands on this region. There are in total six ^i^PrS^–^ and six RCOO^–^ ligands on the polar regions of **SD/Ag84a** and **SD/Ag84b**. On one pole of **SD/Ag84a**, six ^*n*^PrCOO^–^ ligands are equally distributed at the two sides (Fig. 2c), whereas four PhCOO^–^ ligands are located at one side and the other two at another side of one pole of **SD/Ag84b** (Fig. 2d). Moreover, two μ_2_-κ^1^:κ^1^ PhCOO^–^ ligands are adjacent and simultaneously coordinate to the same Ag atom (Ag32), creating a single peak (Ag32) on the pole of **SD/Ag84b**. Although the organic coats are different in **SD/Ag84a** and **SD/Ag84b**, the same silver atom counts suggested that they are cage isomers. The driving force of the isomeric silver nanocluster should be most probably generated from the steric hindrance of different carboxylate groups. Thus, we successfully filled in the blanks in isomeric silver nanoclusters using a soft/hard double-ligand strategy.

Inspired by the isomeric silver skeletons of **SD/Ag84a** and **SD/Ag84b**, we also tried to explore the possibility of conversion between them under the carboxylic acid stimulus. The isomerization experiments were performed in respective reaction mother liquors without removing crystals and by adding a portion of another kind of carboxylic acid. Interestingly, we found that **SD/Ag84b** can be isolated after the synthesis of **SD/Ag84a** by adding bulkier PhCOOH for the second-step reaction; however, the transformation from **SD/Ag84b** to **SD/Ag84a** failed by adding smaller ^*n*^PrCOOH into the system for synthesizing **SD/Ag84b**, which indicated that the bulkier the carboxylic acid, the stronger the inducing effect.

**Fig. 3:**
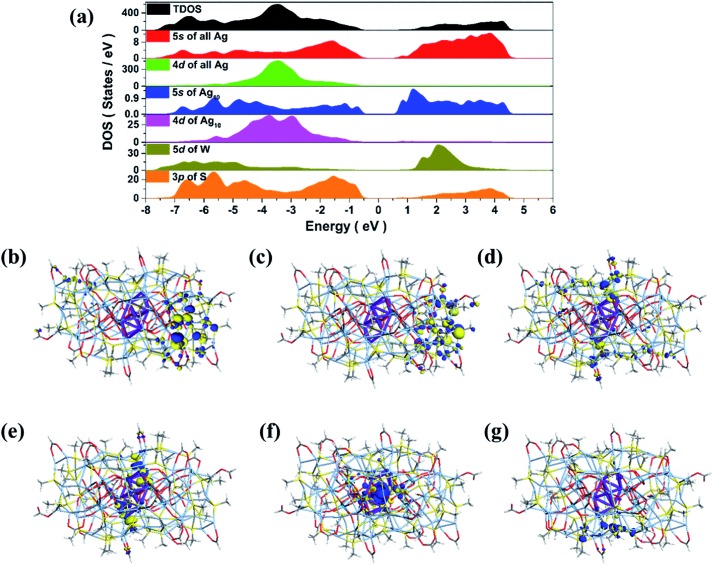
Total DOS and partial DOS of **SD/Ag84a** (a). Frontier molecular orbitals: HOMO–2 (b), HOMO–1 (c), HOMO (d) and LUMO (e), LUMO+1 (f) and LUMO+2 (g).

Considering the subvalent characteristics of the Ag_10_ kernel, we deduced the charge of it to be +4 based on the formula determined by high-quality SCXRD data, which was also further confirmed by the DFT calculations (see details in ESI†). The electronic structure of **SD/Ag84a** (Fig. 3a) demonstrates that both 5s states of the outer Ag_74_ shell and 3p states of S play a pivotal role in the valence band maximum (VBM), while only the 5s states of Ag (especially from the inner Ag_10_ kernel) are dominant in the conduction band minimum (CBM). The resultant band gap of 1.12 eV is comparable with the observed value (*E*_g_ = 1.24 eV) from solid UV-Vis measurement results discussed below. Thus, the corresponding absorption peak can be attributed to electronic transition from the outer Ag_74_ shell to the inner Ag_10_ kernel. Moreover, frontier molecular orbitals (Fig. 3b–g) also indicate that the three highest occupied orbitals (HOMO, HOMO–1 and HOMO–2) are concentrated in the 5s orbitals and 4d orbitals of the outer Ag_74_ shell, while the two lowest unoccupied orbitals (LUMO and LUMO+1) mainly consist of 5s orbitals (derived from octahedron-like Ag_6_) and 4d orbitals (involving two wing-like Ag_2_ units) of the inner Ag_10_ kernel. Therefore, it is concluded that the inner Ag_10_ core features an electron-deficient state, consistent with the +4 valence estimated crystallographically.

### The optical properties of **SD/Ag84a**

**Fig. 4:**
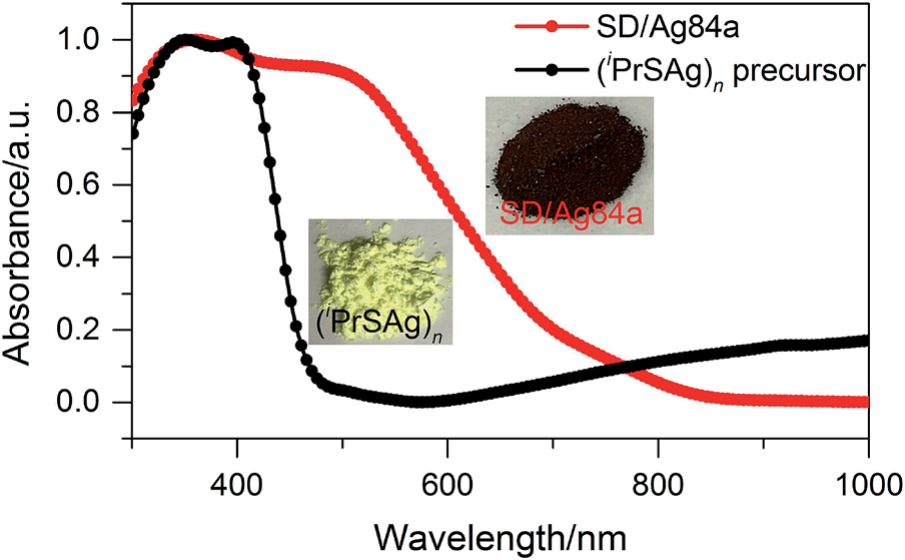
The UV-Vis absorption spectra of **SD/Ag84a** and the (^i^PrSAg)_*n*_ precursor.

The solid state UV-Vis absorption spectrum of **SD/Ag84a** was recorded at room temperature in the wavelength range from 300 to 1000 nm. As depicted in Fig. 4, there is one intense absorption band centered at 361 nm and a shoulder peak at 500 nm, respectively. The high energy absorption centered at 361 nm originates from the n → π* transition of ^i^PrS^–^, as similarly observed in the absorption spectrum of the (^i^PrSAg)_*n*_ precursor. The low energy absorption centered at 500 nm can be attributed to the charge transfer transition from the S 3p to Ag 5s orbitals. Using a transformed Kubelka–Munk plot (Fig. S5†), the HOMO–LUMO gap of **SD/Ag84a** was found to be 1.24 eV, indicating that **SD/Ag84a** is a potential narrow-band-gap semiconductor. By contrast, the optical gap of the (^i^PrSAg)_*n*_ precursor was calculated to be 2.52 eV, which matches with the color of the samples: **SD/Ag84a** is red, whereas the (^i^PrSAg)_*n*_ precursor is yellow (see the insets of Fig. 4).

**Fig. 5:**
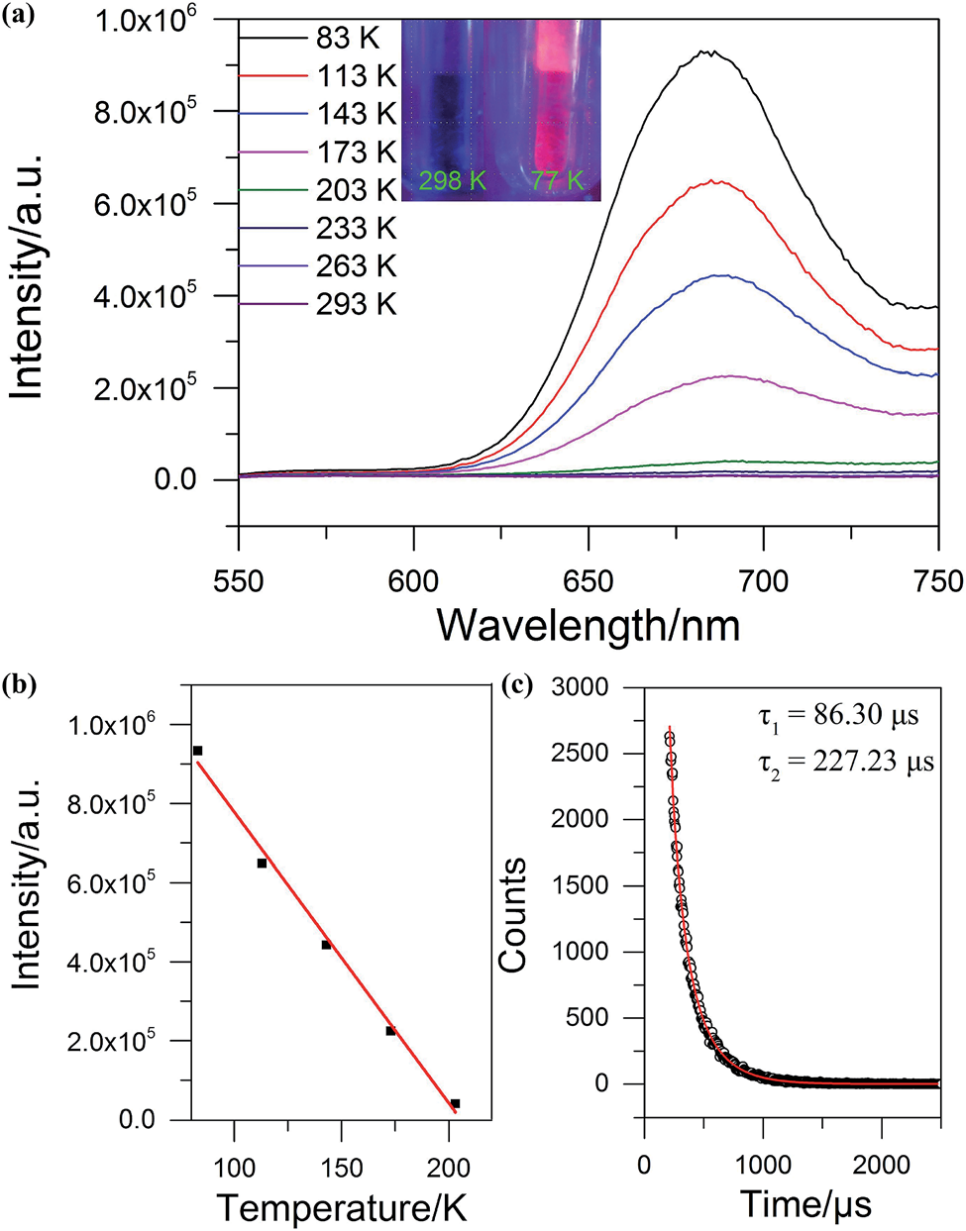
(a) The temperature-dependent emission spectra of **SD/Ag84a** under 468 nm excitation. Insets: the photographs of **SD/Ag84a** irradiated with 365 nm UV light at 298 and 77 K. (b) The plot of temperature *vs.* maximum emission intensity (red line is the linear fitting in the range of 83–203 K). (c) Luminescence lifetime of **SD/Ag84a** recorded at 83 K (red line is the fitting curve).

**SD/Ag84a** does not emit at room temperature in the solid state under a 365 nm hand-held UV light; however, it emits bright-red light at 77 K, which is detectable by the naked eye (see the insets of Fig. 5). The temperature dependent fluorescence spectra were recorded under 468 nm excitation in the temperature range of 293–83 K. From Fig. 5a, we can see that **SD/Ag84a** is almost non-emissive from 293 to 203 K. Upon cooling from 203 to 83 K, **SD/Ag84a** starts emitting with the maximum emission band centered at 689 nm, which is gradually blue-shifted to 685 nm at 83 K along with the increase of luminous intensity. This low-temperature emission should originate from the ligand-to-metal charge transfer (LMCT, charge transfer from S 3p to Ag 5s) perturbed by Ag···Ag interactions.^16^ The temperature dependence behavior should be relevant with variable molecular rigidity and Ag···Ag interactions at different temperatures. The emission intensity was found to be sensitive to temperature and showed good linearity with the corresponding temperature ranging from 83 to 203 K (Fig. 5b), with the linear equation *I*_max_ = 1511090 – 7364.75*T*. The linear equation correlation coefficient is 0.992, which is suitable for temperature detection in the temperature range of 83–203 K. The fluorescence lifetime of **SD/Ag84a** was measured at 83 K (Fig. 5c), with the lifetime value falling on the scale of microseconds (*τ*_1_ = 86.30 μs and *τ*_2_ = 227.23 μs), suggesting a triplet state emission.

## Conclusions

In conclusion, we successfully achieved the assembly of isomeric Ag_84_ nanoclusters for the first time by the combination of anion template and soft/hard double-ligand strategies. The comparative structural analysis indicated that the isomerism mainly occurred on the polar regions of the prolate spheres. The differences are found in both the silver skeleton and ^i^PrS^–^/RCOO^–^ ligand distributions. The driving force for such an isomerism is dominated by the steric hindrance of carboxylates. What’s more interesting is that the Ag_10_ kernel as a grown nanocrystal from the smallest octahedral Ag_6_ unit in face-centred cubic (fcc) silver metal was first identified in the innermost region of the Ag_84_ nanocluster. The results obtained in this study are not only the pioneering results for isomeric silver nanoclusters but also enrich the library of multi-shell silver nanoclusters containing both novel POM templates and ultrasmall reductive silver nanocrystals.

## Conflicts of interest

There are no conflicts to declare.

## Supplementary Material

Supplementary information

Crystal structure data
