# Supplementary material for: Carboxylic acid stimulated silver shell isomerism in a triple core–shell Ag84 nanocluster
Source: Chem Sci. 2019 Mar 29;10(18):4862–7. doi: 10.1039/c8sc05666h (PMC6520922; doi:10.1039/c8sc05666h)
Supplement: Supplementary file 1 [file SC-010-C8SC05666H-s001.pdf]

Electronic Supplementary Material (ESI) for Chemical Science.
This journal is © The Royal Society of Chemistry 2019

Electronic Supporting Information

Carboxylic acid stimulated silver shell isomerism in triple core-shell Ag_{84} nanocluster

Zhi Wang,[a] Hao-Tian Sun,[a] Mohamedally Kurmoo,[c] Qing-Yun Liu,[d] Gui-Lin Zhuang,[*,b] Quan-Qin Zhao,[a] Xing-Po Wang,[a] Chen-Ho Tung,[a] and Di Sun[*, a, b]

[a]Key Laboratory of Colloid and Interface Chemistry, Ministry of Education, School of Chemistry and Chemical Engineering, State Key Laboratory of Crystal Materials, Shandong University, Jinan, 250100, People's Republic of China E-mail: dsun@sdu.edu.cn

[b]College of Chemical Engineering and Materials Science, Zhejiang University of Technology, Hangzhou, 310032, People's Republic of China. E-mail: glzhuang@zjut.edu.cn

[c]Institut de Chimie de Strasbourg, Université de Strasbourg, CNRS-UMR 7177, 4 rue Blaise Pascal, 67008 Strasbourg Cedex, France.

[d]College of Chemical and Environmental Engineering, Shandong University of Science and Technology, Qingdao, 266590, People's Republic of China.

Experiment details

The (^{i}PrSAg)$_n$ precursor was prepared according to the literature.[1] All other chemicals and solvents used in the syntheses were of analytical grade and used without further purification. ^{i}PrSH (Adamas-beta®) was purchased from Shanghai Titan Scientific Co., Ltd. Infrared spectrum was recorded on a PerkinElmer Spectrum Two in the frequency range of 4000-500 cm^{-1}. The elemental analyses (C, H contents) were determined on a Vario EL III analyzer. The diffuse-reflectance spectra were performed on UV−Vis spectrophotometer (Evolution 220, ISA-220 accessory, Thermo Scientific) using a built-in 10 mm silicon photodiode with a 60 mm Spectralon sphere. Powder X-ray diffraction (PXRD) data were collected on a Philips X'Pert Pro MPD X-ray diffractometer with Cu Kα radiation equipped with an X'Celerator detector. Temperature-dependent photoluminescence measurements were carried out in an Edinburgh spectrofluorimeter (F920S) coupled with an Optistat DN cryostat (Oxford Instruments), and the ITC temperature controller and a pressure gauge were used to realize the variable-temperature measurement in the range of 83-293 K. Spectra were collected at different temperatures after a 3 min homoiothermy. Time-resolved photoluminescence lifetime measurements were performed on the same instrument by using a time-correlated single-photon counting technique. Morphology of the samples and elemental composition analyses were measured using an SU-8010 field emission scanning electron microscope (FESEM; Hitachi Ltd., Tokyo, Japan) equipped with an Oxford-Horiba Inca XMax50 energy dispersive X-ray spectroscopy (EDS) attachment (Oxford Instruments Analytical, High Wycombe, England). ^{13}C NMR spectra were recorded in a J. Young NMR tube on Bruker Avance 500 spectrometers. The chemical shifts are reported in parts-per-million (ppm) relative to the residual solvent peak of the deuterated methanol (^{13}C) (δ = 48.80 ppm).

X-ray Crystallography

Single crystals of **SD/Ag84a** and **SD/Ag84b** with appropriate dimensions were chosen under an optical microscope and quickly coated with high vacuum grease (Dow Corning Corporation) to prevent decomposition. Intensity data and cell parameters were recorded at 100 K for **SD/Ag84a** on a Rigaku Oxford Diffraction XtaLAB Synergy diffractometer equipped with a HyPix-6000HE area detector using Mo Kα (λ = 0.71073 Å) from PhotonJet micro-focus X-ray Source. Single-crystal X-ray diffraction data of **SD/Ag84b** was collected at 100 K on synchrotron radiation X-ray diffraction (λ = 0.68877 Å) using BL17B at the Shanghai Synchrotron Radiation Facility (SSRF) (Shanghai, China). The structure was solved using the charge-flipping algorithm, as implemented in the program *SUPERFLIP*[2] and refined by full-matrix least-squares techniques against F_o^2 using the SHELXL program[3] through the OLEX2 interface.[4] Hydrogen atoms at carbon were placed in calculated positions and refined isotropically by using a riding model. Appropriate restraints or constraints were applied to the geometry and the atomic displacement parameters of the atoms in the cluster. All structures were examined using the Addsym subroutine of PLATON[5] to ensure that no additional symmetry could be applied to the models. Pertinent crystallographic data collection and refinement parameters are collated in Table S2. Selected bond lengths and angles are collated in Table S3.

Computational Detail

Geometrical optimizations were conducted by using DMol3 module in the Material Stdudio program.[6,7] Exchange-correlation effect was treated with the generalized gradient approximation (GGA) and the Perdew Burke Ernzerhof (PBE).[8] Both Ag and W atoms were treated by Effective Core Potentials, and the remaining atoms were treated as all electron basic set of DND (Double Numerical plus *d*-functions).[9] In order to reduce computational cost, single-crystal diffraction structure was fully relaxed with some necessary simplification that both butyric acid and propane-2-thiol are simplified as formic acid and methanethiol, respectively. The convergence threshold for the maximum energy change was 2×10^{-5} Ha., and the convergence threshold for the maximum force are 0.004 Hartree $Å^{-1}$. Furthermore, frontier molecular orbitals were also identified.

Moreover, based on the relaxed structure, the density functional states were evaluated by using of VASP program[10] in the theoretical level of PBE[8] functional. Core electrons effect on the valence electron density were represented by using Projector Augmented Wave (PAW) method.[11] The Brillouin zone was sampled by k-points mesh of 1×1×1. The SCF convergence is set to 1×10^{-5} eV.

Synthesis of SD/Ag84a

A mixture of (^{i}PrSAg)$_n$ (0.05 mmol, 9.2 mg), Ag_2O (0.05 mmol, 11.6 mg), Na_2WO_4 (0.02 mmol, 6.6 mg) and $^{n}C_3H_7COOH$ (0.15 mmol, 14 μL) was dissolved in the mixed solvents of MeOH/DMF (5 mL, v/v = 4/1), then the mixture was sealed into 25 mL Teflon-lined autoclave under autogenous pressure and heated at 75 ^{o}C for 2000 min. After cooling to room temperature, the orange solution was filtrated and evaporated in the dark, red block crystals were isolated with a yield of 20 %. Elemental analyses calc. (found) for **SD/Ag84a** ($C_{194}H_{412}Ag_{84}O_{90}S_{42}W_{14}$): C, 13.57 (13.49); H, 2.42 (2.38) %. Selected IR peaks (cm^{-1}): 3687 (w), 2951 (m), 1541 (s), 1453 (m), 1394 (s), 1305 (m), 1242 (m), 1144 (m), 1051 (s), 1030 (s), 1012 (m), 886 (m), 814 (s), 653 (s), 591 (s).

Synthesis of SD/Ag84b

The synthesis conditions were similar to those described for **SD/Ag84a**, except using PhCOOAg (0.1 mmol, 22.9 mg) instead of Ag_2O and $^{n}C_3H_7COOH$, the mixture were heated 65 ^{o}C for 2000 min, after cooling to room temperature, red rhombus crystals were isolated with a yield of 3 %. Elemental analyses calc. (found) for **SD/Ag84b** ($C_{246}H_{370}Ag_{84}O_{88}S_{42}W_{14}$): C, 16.68 (16.61); H, 2.11 (2.07) %.

SD/Ag84b can also be isolated by adding PhCOOH (0.11 mmol, 0.0134g) to the reaction mixture after the synthesis of **SD/Ag84a**, then this mixture was again sealed into 25 mL Teflon-lined autoclave and heated at 75 ^{o}C for 1200 min. After cooling to room temperature, red rhombus crystals were isolated with a yield of 3 %.

Figure S1: The binding mode of $(W_7O_{26})^{10-}$ towards silver atoms in SD/Ag84a.

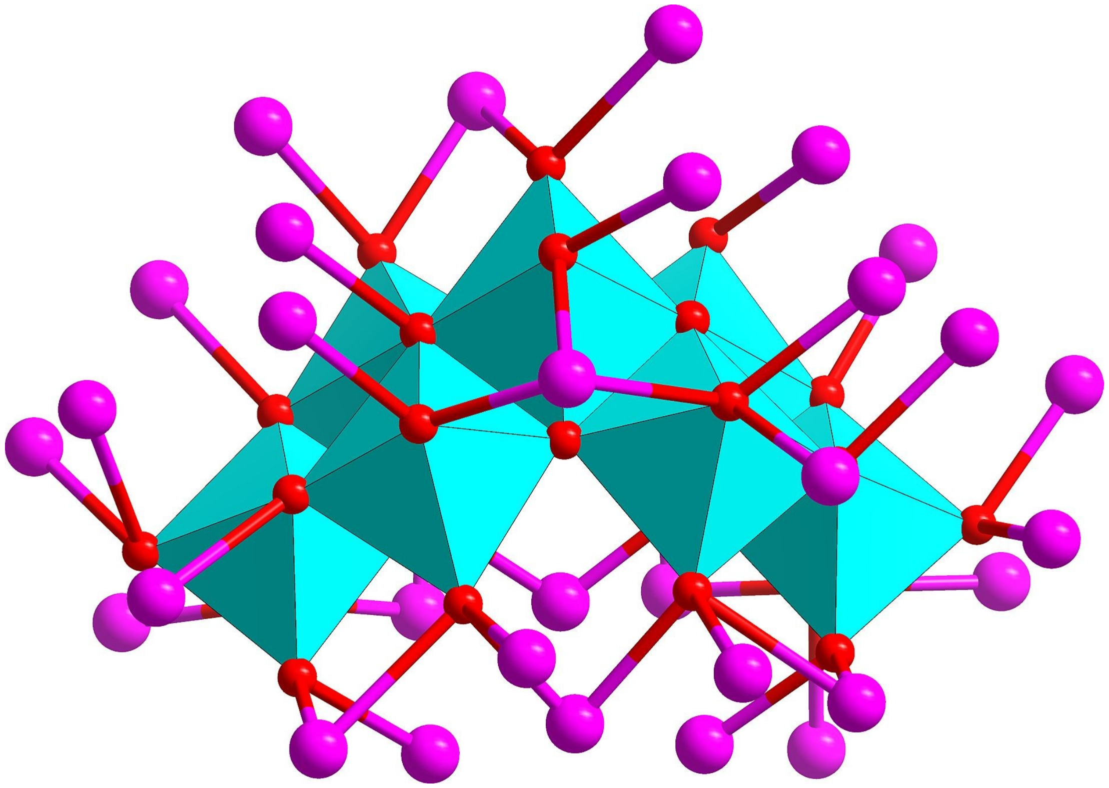

Figure S2: The μ_6 coordination mode of S^{2-} towards silver shell in SD/Ag84a, with Ag-S bond are highlighted by black thick style.

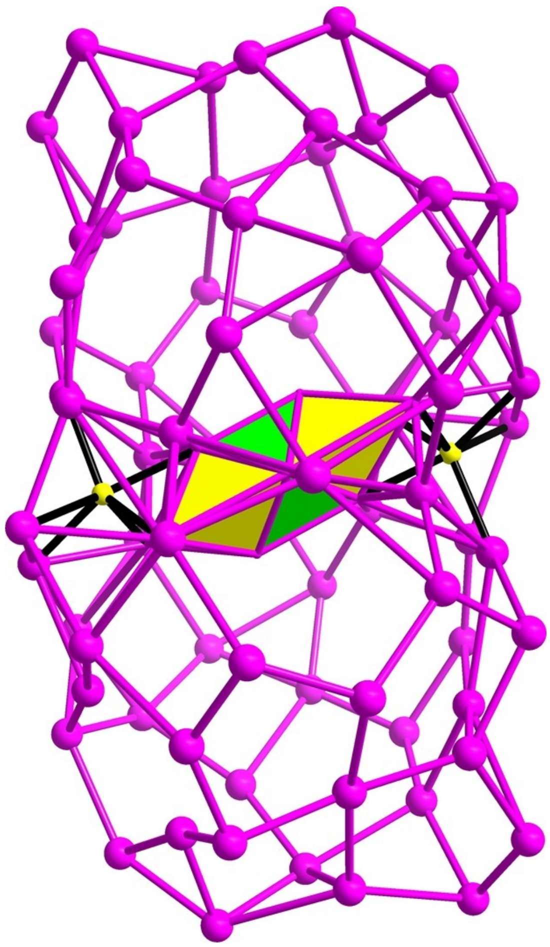

Figure S3：13C NMR of HCl digested reaction mother solution for SD/Ag84a.

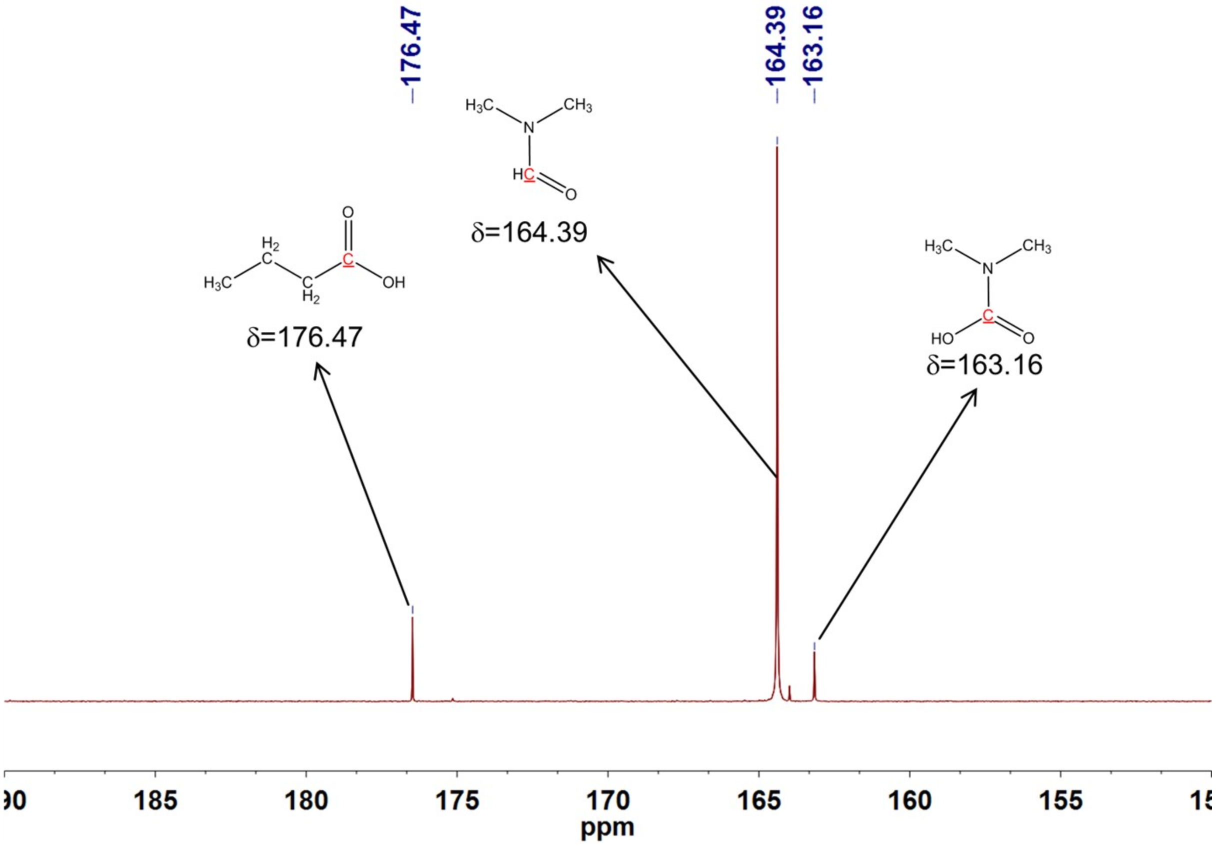

Figure S4：^{13}C NMR of HCl digested reaction mother solution for SD/Ag84b.

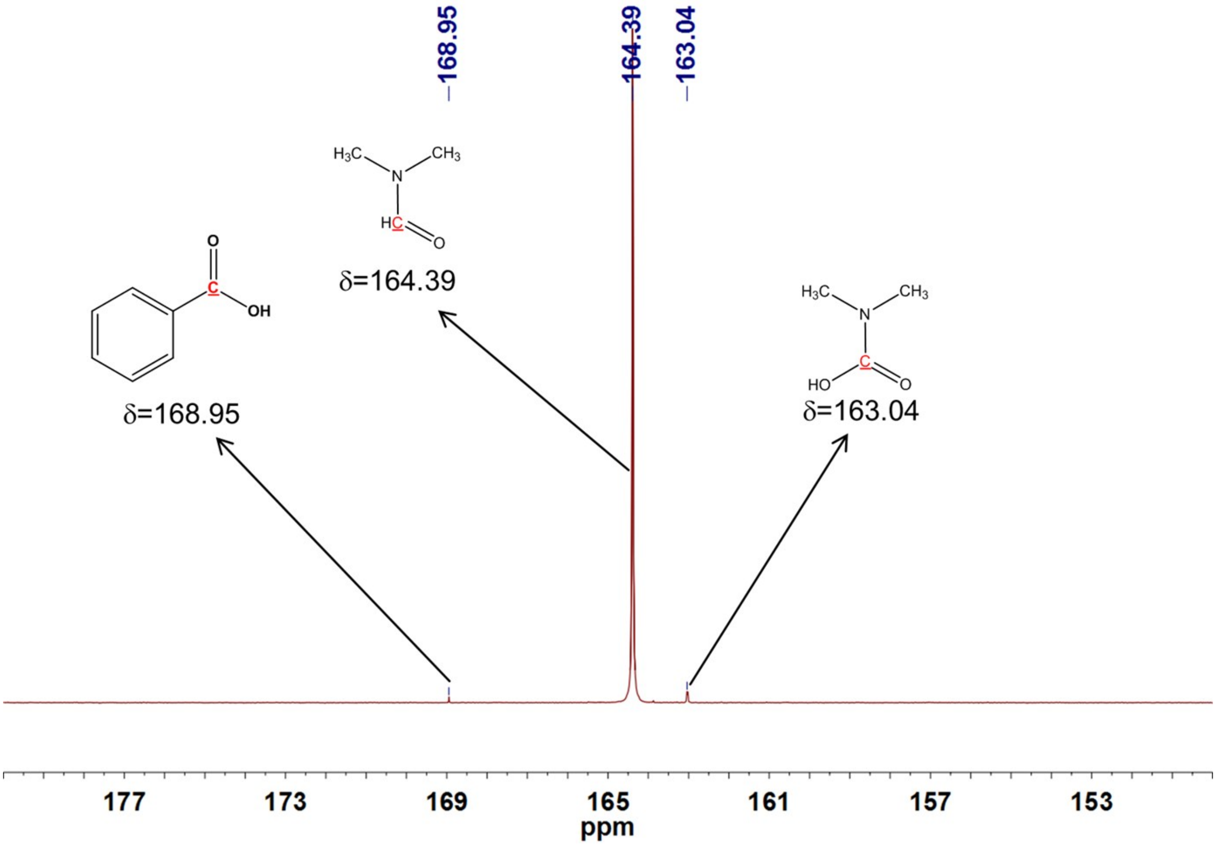

Figure S5: Diffuse reflectance spectra of SD/Ag84a and (^{i}PrSAg)$_n$ and extrapolation of the edge of the Kubelka-Munk function.

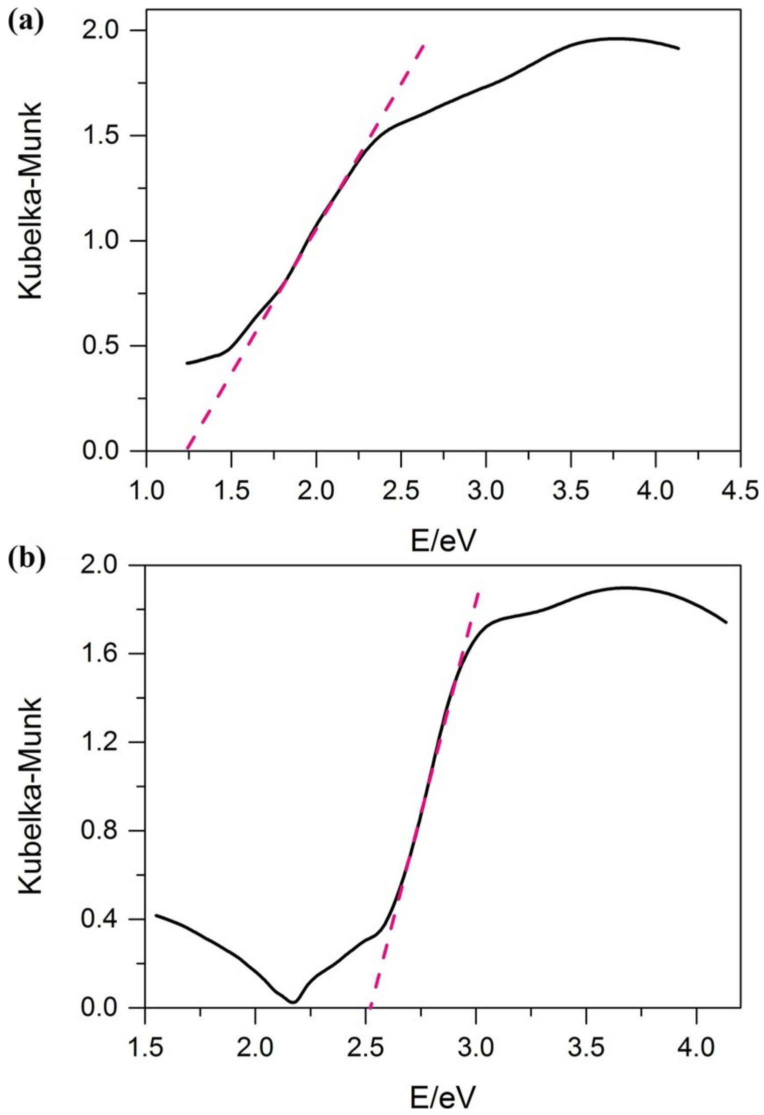

Figure S6: The IR spectrum of SD/Ag84a.

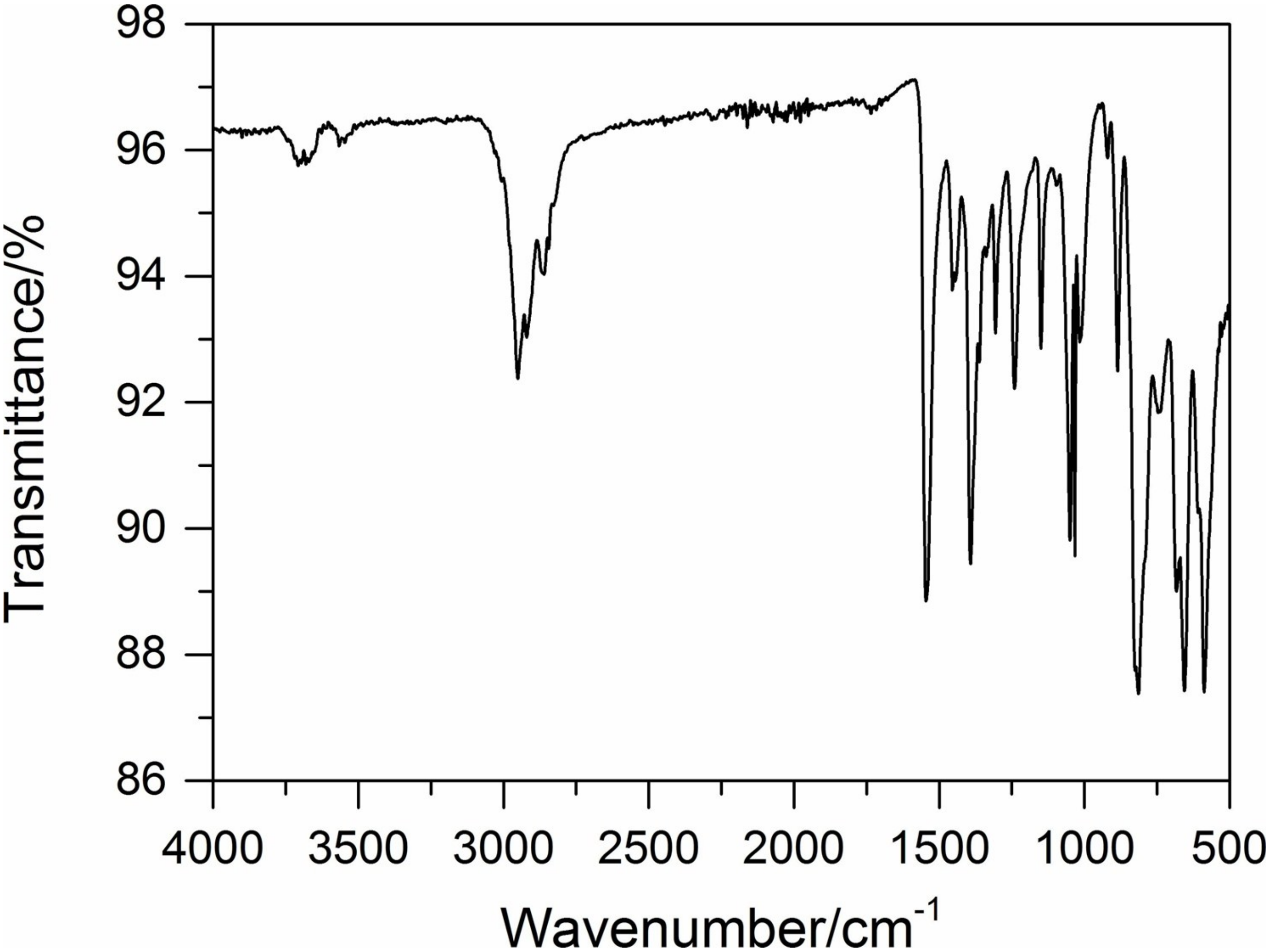

Figure S7: Microscope photograph of crystals of SD/Ag84a.

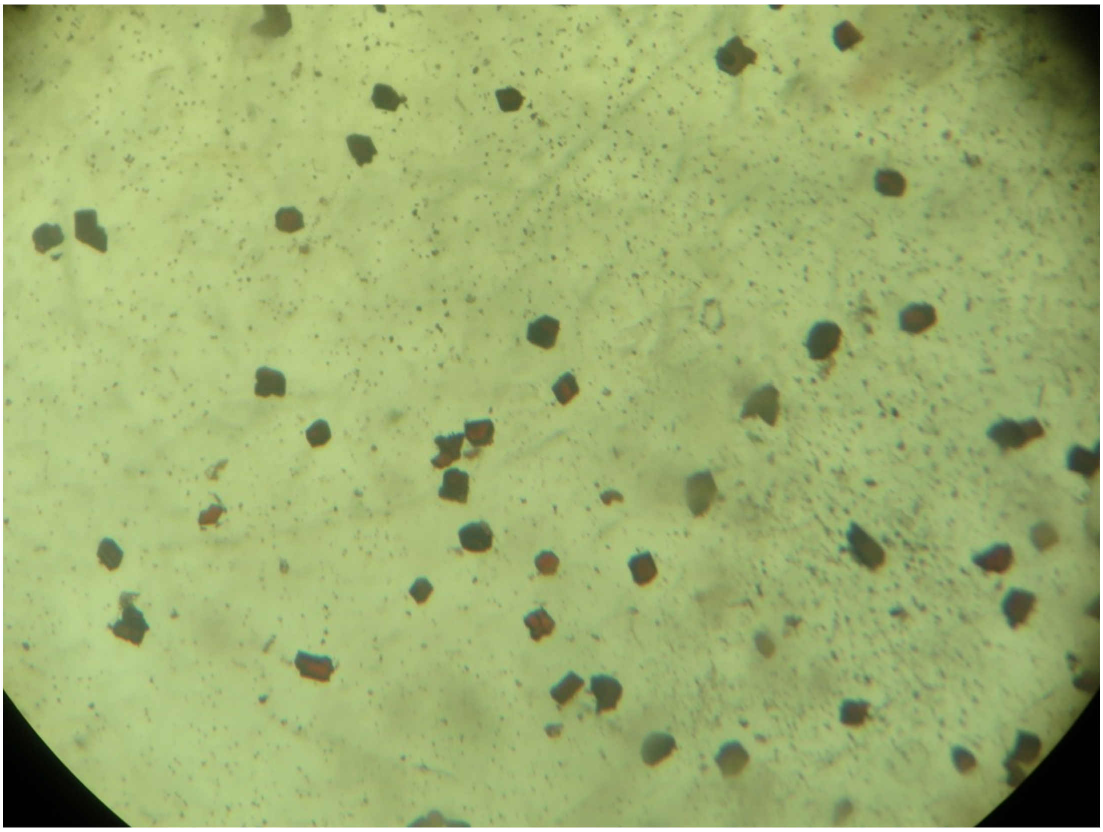

Figure S8: Molecules packing diagrams in 2×2×2 unit cell of SD/Ag84a viewed from different directions.

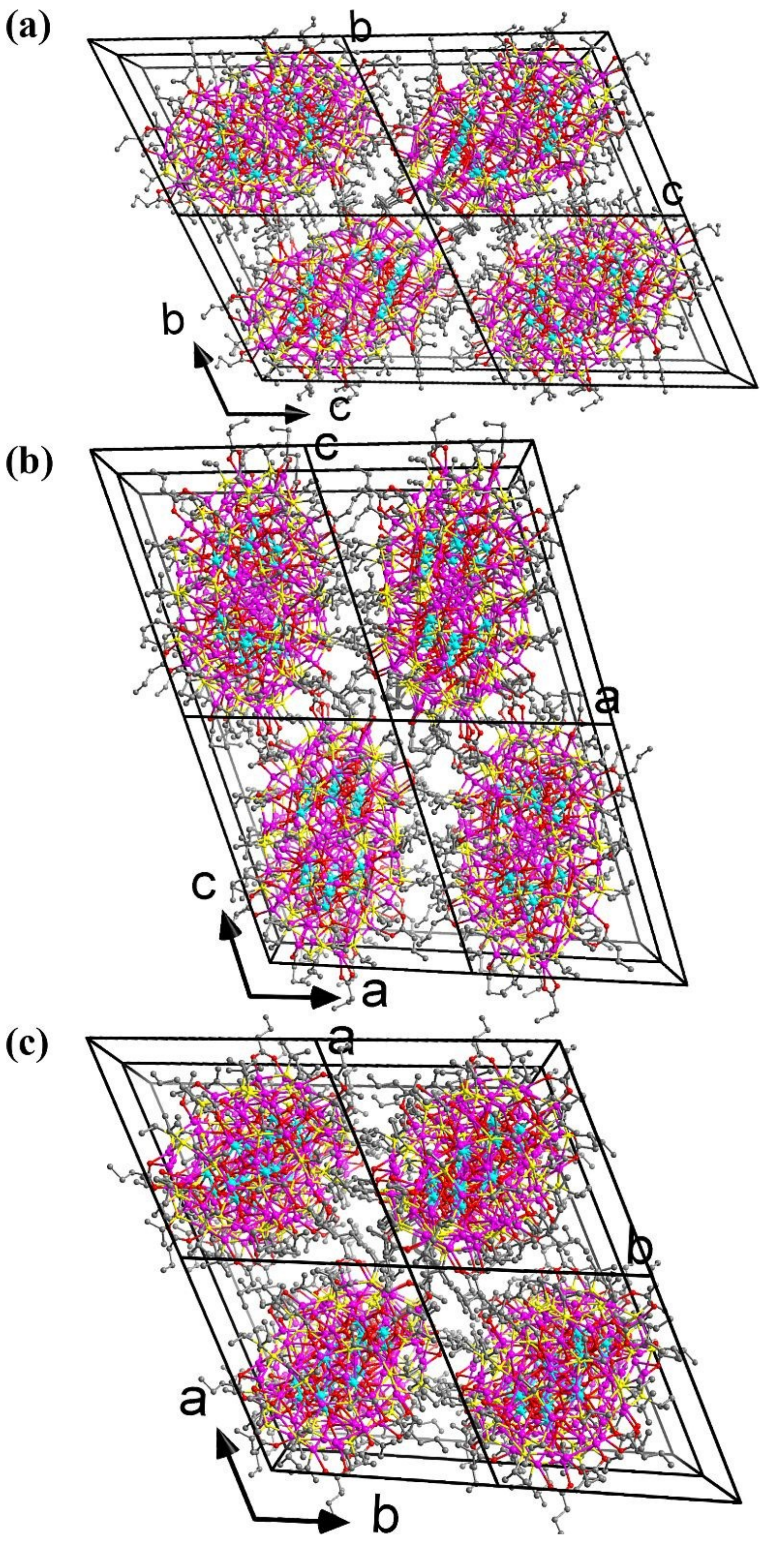

Figure S9: Molecules packing diagrams in 2×2×2 unit cell of SD/Ag84b from different directions.

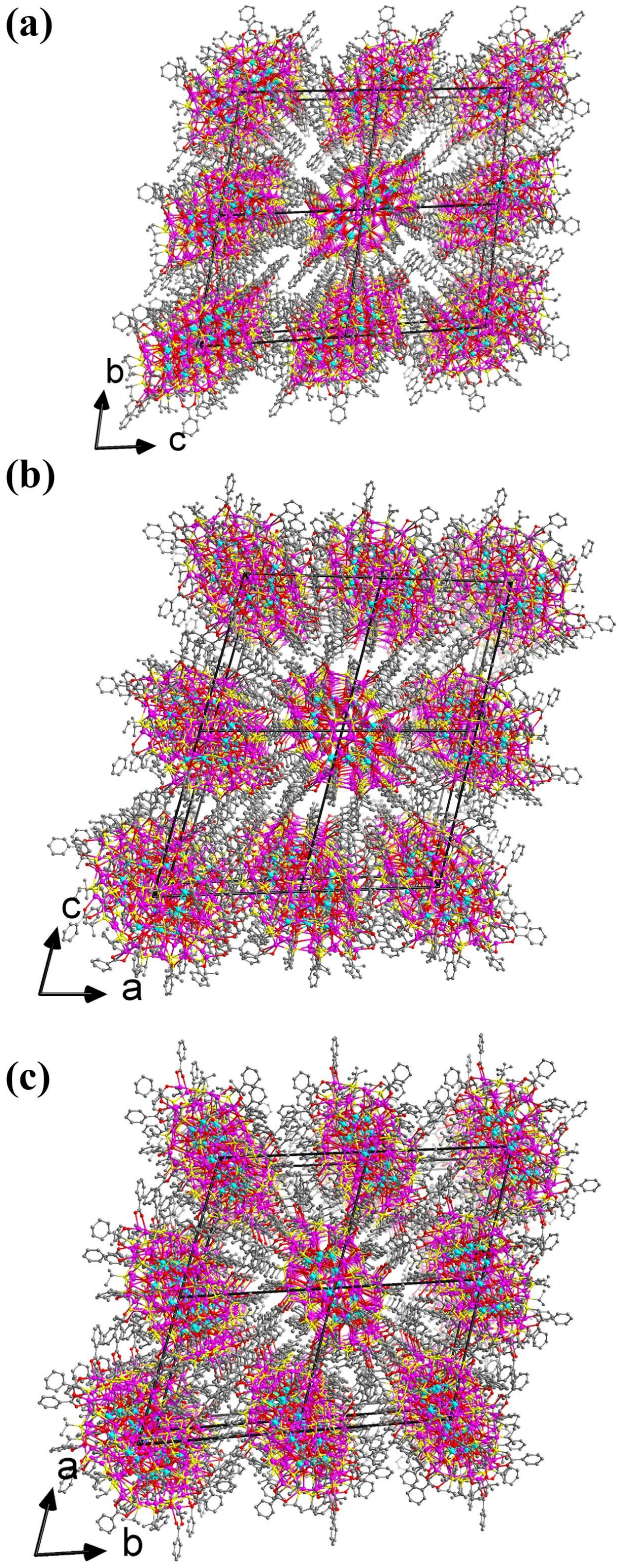

Figure S10: SEM and elemental mapping images of SD/Ag84a.

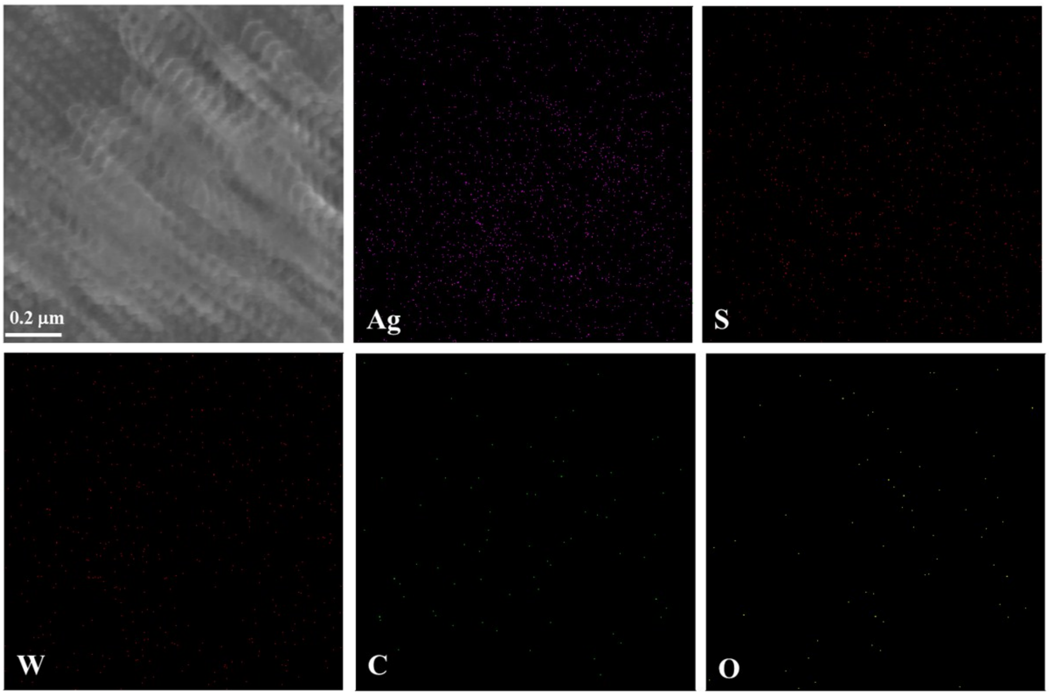

Figure S11: SEM and elemental mapping images of SD/Ag84b.

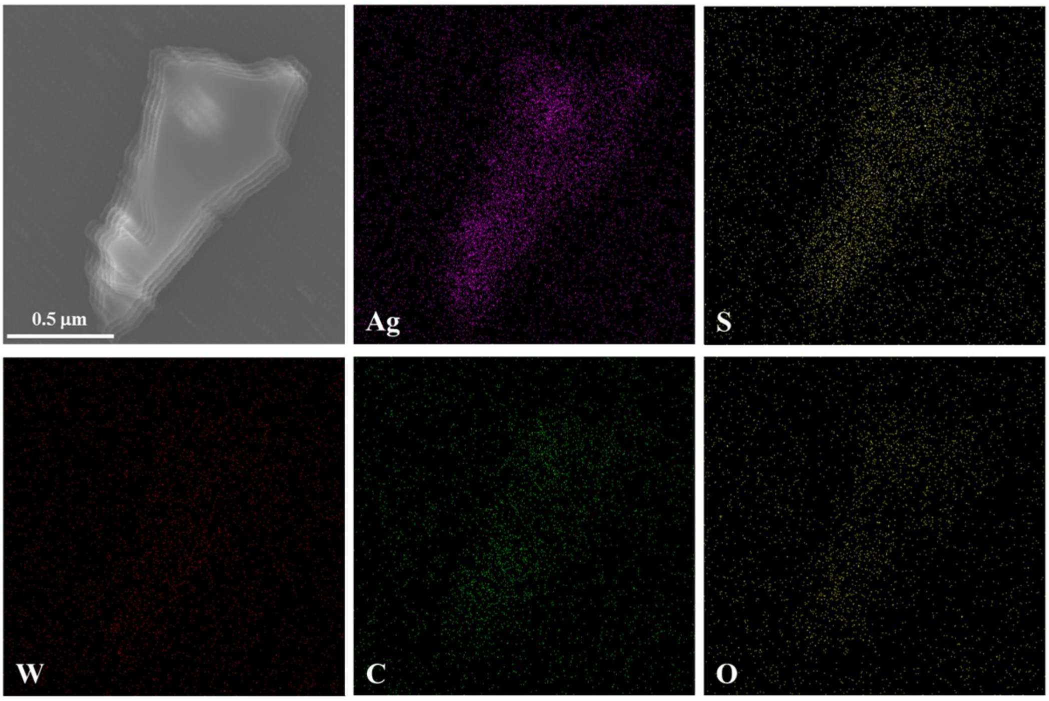

Figure S12: Compared PXRD patterns of SD/Ag84a.

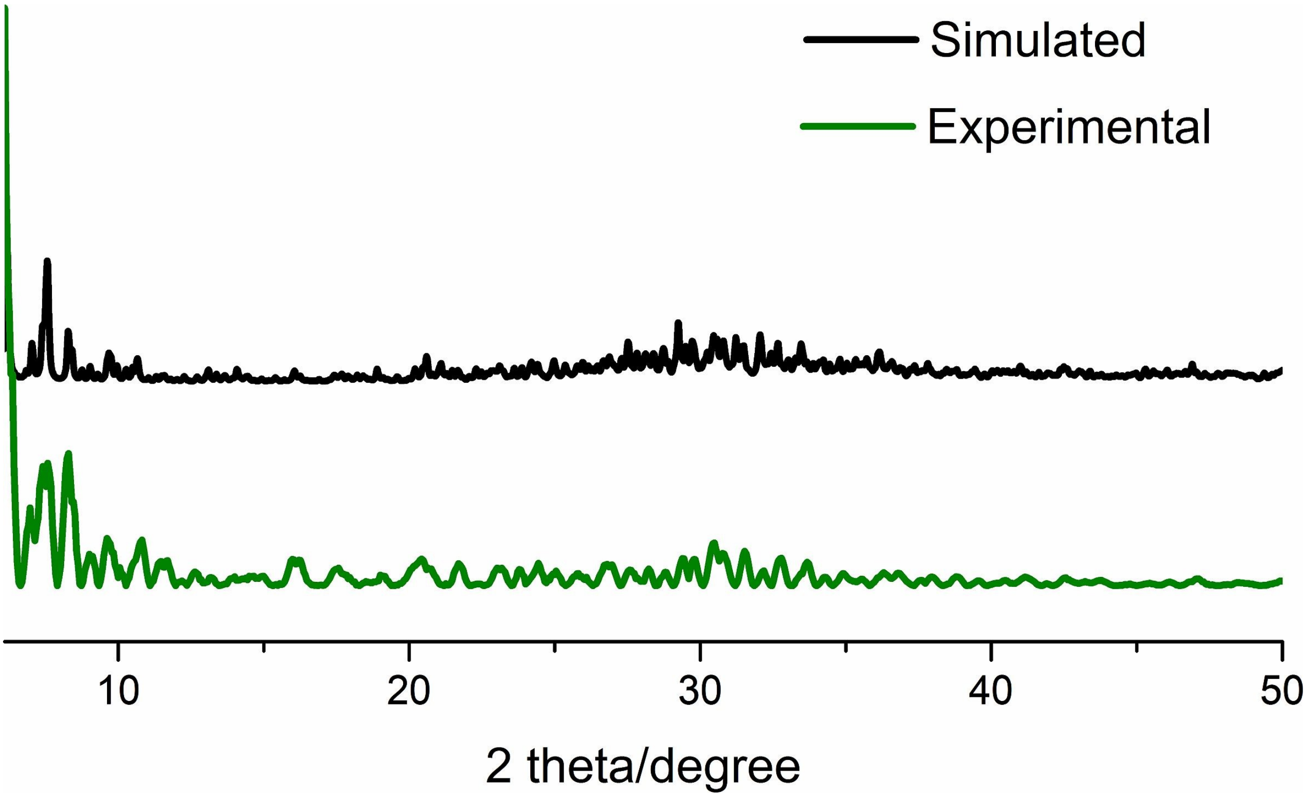

Figure S13: Compared PXRD patterns of SD/Ag84b.

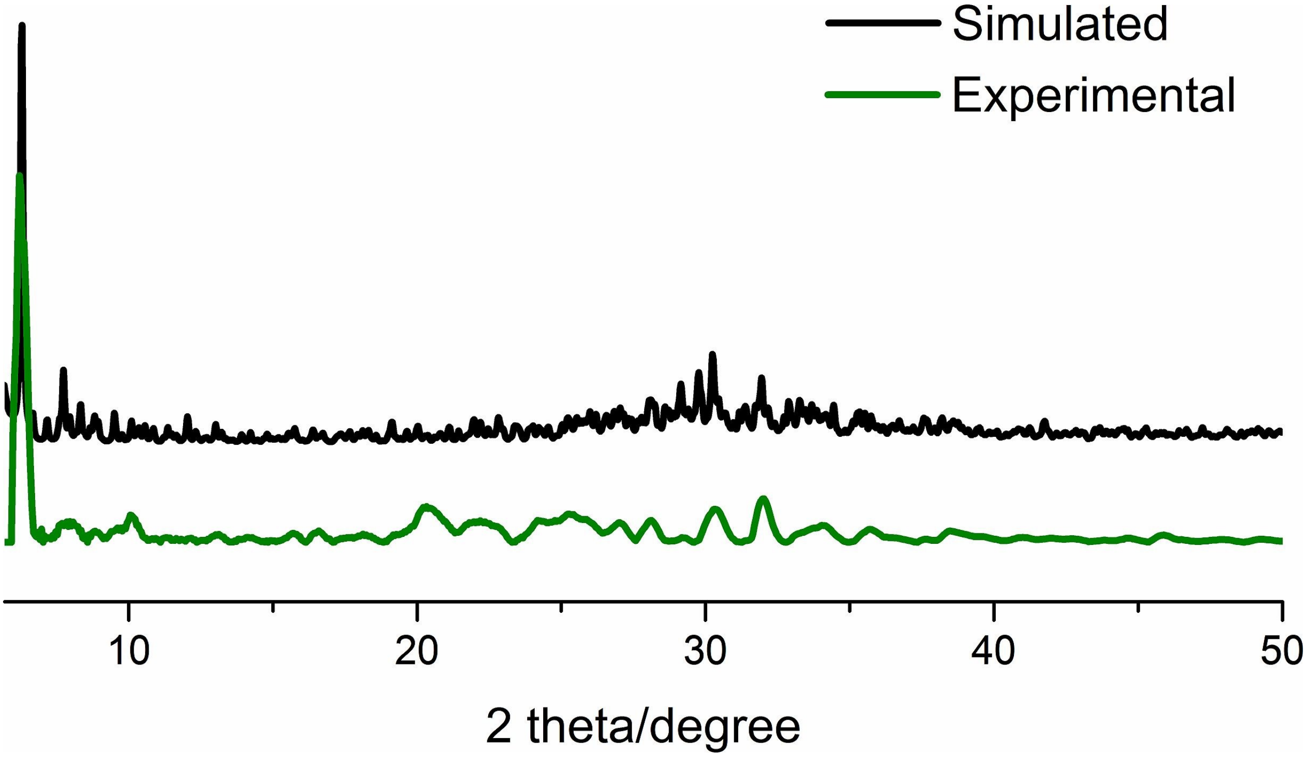

Table S1: Comparative Geometry Parameters for SD/Ag84a and SD/Ag84b.

	**SD/Ag84a**	**SD/Ag84b**
Ag···Ag distances in Ag_6 / Å	2.732-2.915	2.741-2.913
Ag···Ag distances in four caps on Ag_6 / Å	2.982-3.408	2.962-3.411
Ag···Ag distances in Ag_{74} / Å	2.812-3.417	2.614-3.353
Ag-S_{ligand} / Å	2.352-2.814	2.158-2.949
Ag-$S_{sulfide}$ / Å	2.394-2.776	2.374-2.658
Ag-$O_{carboxylate}$ / Å	2.195-2.688	2.189-2.764
Ag-O_{POM} / Å	2.170-2.773	2.147-2.798
W-O / Å	1.735-2.282	1.737-2.255
Coordination mode of $^{i}PrS^{-}$	14 μ_3, 26 μ_4	16 μ_3, 22 μ_4, 2 μ_5
Coordination mode of $RCOO^{-}$	14 μ_2-κ^1:κ^1<br>4 μ_3-κ^2:κ^1	14 μ_2-κ^1:κ^1<br>4 μ_3-κ^2:κ^1
Coordinated Ag atoms on $(W_7O_{26})^{10-}$	32	34

Table S2: Crystal Data Collection and Structure Refinement for SD/Ag84a and SD/Ag84b.

Compound	**SD/Ag84a**	**SD/Ag84b**
Empirical formula	$C_{194}H_{414}Ag_{84}O_{90}S_{42}W_{14}$	$C_{246}H_{370}Ag_{84}O_{88}S_{42}W_{14}$
X-ray diffractometer	Rigaku Oxford Diffraction XtaLAB Synergy	SSRF beamline BL17B
Formula weight	17168.77	17716.90
Temperature/K	99.99(10)	100(2)
Crystal system	triclinic	triclinic
Space group	*P*-1	*P*-1
a/Å	20.2607(2)	21.5617(11)
b/Å	20.6711(2)	22.2473(10)
c/Å	26.2425(3)	24.5285(13)
α/°	109.7140(10)	74.1307(13)
β/°	100.1000(10)	70.9758(13)
γ/°	109.0090(10)	68.5642(17)
Volume/Å^3	9266.29(18)	10193.5(9)
Z	1	1
ρ_{calc}g/cm^3	3.076	2.886
μ/mm^{-1}	8.936	6.444
F(000)	7952.0	8206.0
Radiation	MoKα (λ = 0.71073)	synchrotron (λ = 0.68877)
Reflections collected	96913	126316
Independent reflections	32790 [R_{int} = 0.0295, R_{sigma} = 0.0270]	33781 [R_{int} = 0.0568, R_{sigma} = 0.0516]
Data/parameters	32790/2033	33781/1944
Goodness-of-fit on F^2	1.041	1.027
Final R indexes [I>=2σ (I)]	R_1 = 0.0578, wR_2 = 0.1548	R_1 = 0.0752, wR_2 = 0.1690
Final R indexes [all data]	R_1 = 0.0637, wR_2 = 0.1596	R_1 = 0.0975, wR_2 = 0.1919
Largest diff. peak/hole / e Å^{-3}	3.94/-1.79	5.49/-3.25

Table S3: Selected bond distances (Å) and angles (°) for SD/Ag84a and SD/Ag84b.

SD/Ag84a			
Ag1—Ag8	2.946 (5)	Ag19—S15	2.705 (4)
Ag1—Ag10	3.247 (6)	Ag20—O33	2.27 (2)
Ag1—Ag20	3.096 (5)	Ag20—S11	2.557 (4)
Ag1—O36	2.56 (4)	Ag20—S14	2.494 (4)
Ag1—S6	2.435 (6)	Ag21—Ag22	3.063 (2)
Ag1—S14	2.587 (8)	Ag21—O11	2.520 (8)
Ag2—Ag29	2.933 (4)	Ag21—S12	2.405 (5)
Ag2—$S4^{i}$	2.700 (3)	Ag21—S15	2.400 (5)
Ag2—S9	2.495 (6)	Ag22—Ag40	2.822 (2)
Ag2—S21	2.471 (6)	Ag22—O28	2.256 (19)
Ag3—Ag9	2.881 (3)	Ag22—S12	2.611 (5)
Ag3—Ag29	2.9375 (17)	Ag22—S13	2.373 (6)
Ag3—O1	2.518 (9)	Ag23—Ag24	3.0860 (17)
Ag3—O29	2.280 (12)	Ag23—Ag40	3.1737 (19)
Ag3—S16	2.665 (4)	Ag23—O19	2.390 (8)
Ag3—S21	2.626 (4)	Ag23—O26	2.405 (15)
Ag4—Ag21	3.207 (2)	Ag23—S8	2.468 (4)
Ag4—O19	2.450 (8)	Ag23—S10	2.501 (4)
Ag4—S12	2.468 (4)	Ag24—O25	2.217 (14)
Ag4—S21	2.533 (4)	Ag24—S8	2.603 (4)
Ag5—Ag11	2.944 (2)	Ag24—S9	2.442 (4)
Ag5—O31	2.411 (14)	Ag25—Ag26	3.3546 (13)
Ag5—O40	2.534 (9)	Ag25—$Ag31^{i}$	3.3781 (18)
Ag5—S16	2.523 (5)	Ag25—$Ag42^{i}$	2.9201 (16)
Ag5—S19	2.523 (4)	Ag25—$O4^{i}$	2.522 (8)
Ag6—Ag17	3.154 (3)	Ag25—O24	2.244 (8)
Ag6—Ag41	3.308 (3)	Ag25—S9	2.409 (4)
Ag6—O23	2.562 (8)	Ag26—Ag27	2.7317 (11)
Ag6—S1	2.454 (4)	Ag26—$Ag27^{i}$	2.7470 (11)
Ag6—$S19^{i}$	2.382 (5)	Ag26—$Ag28^{i}$	2.7487 (12)
Ag7—Ag16	3.070 (2)	Ag26—Ag28	2.7806 (12)
Ag7—Ag41	2.908 (2)	Ag26—Ag39	3.3574 (12)
Ag7—O44	2.273 (13)	Ag26—$O5^{i}$	2.307 (8)
Ag7—S7	2.558 (5)	Ag26—O24	2.301 (8)
Ag7—S8	2.573 (4)	Ag27—Ag28	2.8296 (11)
Ag8—Ag20	2.8120 (17)	Ag27—$Ag28^{i}$	2.9155 (11)
Ag8—Ag34	3.054 (3)	Ag27—Ag39	3.0578 (12)
Ag8—O12	2.473 (9)	Ag27—Ag42	3.0058 (12)
Ag8—O16	2.464 (9)	Ag27—O8	2.294 (8)

Ag8—S6	2.441 (4)	Ag27—O17	2.312 (7)
Ag8—S11	2.524 (4)	Ag28—Ag39[i]	2.9819 (12)
Ag9—Ag11	2.836 (2)	Ag28—Ag42[i]	3.0235 (12)
Ag9—Ag21	2.884 (3)	Ag28—O10	2.515 (8)
Ag9—O30	2.243 (11)	Ag28—O20	2.380 (8)
Ag9—S15	2.446 (5)	Ag28—S4[i]	2.553 (3)
Ag9—S16	2.595 (5)	Ag29—Ag37[i]	3.1191 (16)
Ag10—Ag12	3.072 (2)	Ag29—S3[i]	2.429 (4)
Ag10—Ag18	3.272 (2)	Ag29—S21	2.410 (4)
Ag10—Ag32	3.077 (2)	Ag30—S19	2.397 (5)
Ag10—O36	2.20 (4)	Ag30—S20	2.428 (5)
Ag10—S5	2.522 (4)	Ag31—S18	2.387 (5)
Ag10—S6	2.653 (5)	Ag31—S20	2.384 (4)
Ag11—Ag13	3.1295 (19)	Ag32—S5	2.352 (4)
Ag11—O2	2.522 (8)	Ag32—S18	2.364 (5)
Ag11—S16	2.467 (4)	Ag33—S10	2.560 (4)
Ag11—S17	2.442 (4)	Ag33—S11	2.510 (5)
Ag12—O15	2.479 (8)	Ag33—S13	2.490 (6)
Ag12—S2	2.481 (4)	Ag34—Ag35	2.9346 (17)
Ag12—S6	2.500 (4)	Ag34—O16	2.425 (9)
Ag13—Ag18	3.299 (2)	Ag34—O38	2.323 (14)
Ag13—O32	2.221 (13)	Ag34—S7	2.660 (4)
Ag13—O40	2.358 (9)	Ag34—S11	2.525 (4)
Ag13—S17	2.471 (4)	Ag35—Ag36	3.3533 (19)
Ag13—S18	2.771 (5)	Ag35—Ag41	2.882 (2)
Ag14—Ag18	3.215 (2)	Ag35—O37	2.257 (16)
Ag14—Ag19	3.094 (2)	Ag35—S1	2.576 (4)
Ag14—O6	2.509 (9)	Ag35—S7	2.516 (5)
Ag14—S14	2.542 (4)	Ag36—S1	2.389 (4)
Ag14—S15	2.814 (5)	Ag36—S2	2.368 (4)
Ag14—S17	2.509 (4)	Ag37—Ag38	2.9004 (16)
Ag15—Ag23	2.8946 (17)	Ag37—Ag39	3.2687 (14)
Ag15—Ag33	3.342 (2)	Ag37—O41	2.273 (11)
Ag15—Ag34	2.9721 (17)	Ag37—S2	2.532 (4)
Ag15—O18	2.402 (7)	Ag37—S3	2.591 (4)
Ag15—S7	2.482 (4)	Ag37—S4	2.776 (3)
Ag15—S10	2.520 (4)	Ag38—Ag42	2.9535 (14)
Ag16—Ag24	2.933 (2)	Ag38—O42	2.299 (10)
Ag16—Ag25	3.0374 (18)	Ag38—S4	2.519 (3)
Ag16—S8	2.434 (4)	Ag38—S5	2.449 (4)
Ag16—S20[i]	2.429 (4)	Ag39—O15	2.446 (8)
Ag17—Ag29[i]	2.955 (2)	Ag39—O23	2.170 (8)
Ag17—Ag39	3.1393 (16)	Ag39—S4	2.394 (3)

Ag17—O5^{i}	2.136 (8)	Ag40—O26	2.428 (15)
Ag17—S3	2.421 (4)	Ag40—O27	2.280 (17)
Ag18—Ag32	2.9791 (19)	Ag40—S10	2.615 (4)
Ag18—O35	1.77 (4)	Ag40—S12	2.537 (4)
Ag18—S17	2.614 (4)	Ag41—O39	2.342 (9)
Ag18—S18	2.642 (5)	Ag41—O43	2.195 (12)
Ag19—Ag20	2.964 (2)	Ag41—S1	2.493 (4)
Ag19—Ag21	3.377 (2)	Ag42—O4	2.218 (9)
Ag19—Ag33	3.258 (2)	Ag42—O7	2.400 (9)
Ag19—S13	2.439 (6)	Ag42—S4	2.404 (3)
Ag19—S14	2.474 (5)		
O36—Ag1—S14	112.7 (9)	S14—Ag19—S15	105.19 (15)
S6—Ag1—O36	90.1 (9)	O33—Ag20—S11	100.5 (7)
S6—Ag1—S14	133.5 (3)	O33—Ag20—S14	118.4 (7)
S9—Ag2—S4^{i}	98.08 (15)	S14—Ag20—S11	138.02 (13)
S21—Ag2—S4^{i}	118.62 (19)	S12—Ag21—O11	84.7 (2)
S21—Ag2—S9	143.14 (15)	S15—Ag21—O11	101.8 (2)
O1—Ag3—S16	85.4 (2)	S15—Ag21—S12	172.78 (14)
O1—Ag3—S21	76.5 (2)	O28—Ag22—S12	110.9 (8)
O29—Ag3—O1	125.7 (4)	O28—Ag22—S13	125.8 (8)
O29—Ag3—S16	105.3 (4)	S13—Ag22—S12	123.23 (17)
O29—Ag3—S21	99.7 (4)	O19—Ag23—O26	92.4 (5)
S21—Ag3—S16	154.64 (13)	O19—Ag23—S8	99.5 (2)
O19—Ag4—S12	122.6 (2)	O19—Ag23—S10	108.6 (2)
O19—Ag4—S21	107.9 (2)	O26—Ag23—S8	108.7 (4)
S12—Ag4—S21	122.99 (14)	O26—Ag23—S10	102.3 (4)
O31—Ag5—O40	91.7 (4)	S8—Ag23—S10	136.74 (13)
O31—Ag5—S16	113.4 (4)	O25—Ag24—S8	101.4 (4)
O31—Ag5—S19	99.1 (4)	O25—Ag24—S9	126.2 (4)
S16—Ag5—O40	121.8 (2)	S9—Ag24—S8	130.00 (14)
S19—Ag5—O40	96.9 (2)	O24—Ag25—O4^{i}	87.2 (3)
S19—Ag5—S16	126.6 (2)	O24—Ag25—S9	137.5 (2)
S1—Ag6—O23	91.4 (2)	S9—Ag25—O4^{i}	135.0 (2)
S19^{i}—Ag6—O23	108.3 (2)	O24—Ag26—O5^{i}	78.2 (3)
S19^{i}—Ag6—S1	149.53 (17)	O8—Ag27—O17	104.7 (3)
O44—Ag7—S7	128.4 (4)	O10—Ag28—S4^{i}	94.14 (19)
O44—Ag7—S8	99.3 (4)	O20—Ag28—O10	78.7 (3)
S7—Ag7—S8	119.10 (16)	O20—Ag28—S4^{i}	96.5 (2)
O12—Ag8—S11	77.2 (2)	S21—Ag29—S3^{i}	163.10 (13)
O16—Ag8—O12	81.5 (3)	S19—Ag30—S20	154.97 (15)
O16—Ag8—S11	98.0 (2)	S20—Ag31—S18	162.09 (16)
S6—Ag8—O12	122.6 (3)	S5—Ag32—S18	168.49 (15)
S6—Ag8—O16	92.8 (2)	S11—Ag33—S10	122.49 (14)

S6—Ag8—S11	158.83 (17)	S13—Ag33—S10	114.34 (17)
O30—Ag9—S15	115.9 (3)	S13—Ag33—S11	121.86 (17)
O30—Ag9—S16	102.3 (3)	O16—Ag34—S7	94.5 (2)
S15—Ag9—S16	136.44 (15)	O16—Ag34—S11	99.0 (2)
O36—Ag10—S5	139.6 (12)	O38—Ag34—O16	116.7 (6)
O36—Ag10—S6	93.0 (10)	O38—Ag34—S7	104.4 (5)
S5—Ag10—S6	115.93 (14)	O38—Ag34—S11	106.1 (4)
S16—Ag11—O2	77.8 (2)	S11—Ag34—S7	136.31 (14)
S17—Ag11—O2	110.2 (2)	O37—Ag35—S1	98.9 (4)
S17—Ag11—S16	169.42 (14)	O37—Ag35—S7	123.4 (5)
O15—Ag12—S2	81.8 (2)	S7—Ag35—S1	129.16 (14)
O15—Ag12—S6	121.4 (2)	S2—Ag36—S1	171.92 (13)
S2—Ag12—S6	132.79 (13)	O41—Ag37—S2	111.4 (3)
O32—Ag13—O40	108.5 (4)	O41—Ag37—S3	121.0 (3)
O32—Ag13—S17	134.7 (4)	O41—Ag37—S4	106.6 (3)
O32—Ag13—S18	104.4 (4)	S2—Ag37—S3	117.69 (12)
O40—Ag13—S17	110.0 (2)	S2—Ag37—S4	103.61 (10)
O40—Ag13—S18	84.7 (2)	S3—Ag37—S4	92.06 (10)
S17—Ag13—S18	102.07 (14)	O42—Ag38—S4	107.7 (3)
O6—Ag14—S14	86.7 (2)	O42—Ag38—S5	104.3 (3)
O6—Ag14—S15	132.3 (2)	S5—Ag38—S4	144.24 (12)
O6—Ag14—S17	106.1 (2)	O23—Ag39—O15	82.6 (3)
S14—Ag14—S15	100.32 (15)	O23—Ag39—S4	161.8 (2)
S17—Ag14—S14	139.13 (14)	S4—Ag39—O15	114.9 (2)
S17—Ag14—S15	98.86 (14)	O26—Ag40—S10	98.5 (4)
O18—Ag15—S7	110.1 (2)	O26—Ag40—S12	96.2 (4)
O18—Ag15—S10	111.2 (2)	O27—Ag40—O26	117.9 (8)
S7—Ag15—S10	138.57 (12)	O27—Ag40—S10	114.7 (7)
S20^{i}—Ag16—S8	152.97 (14)	O27—Ag40—S12	118.2 (6)
O5^{i}—Ag17—S3	167.5 (2)	S12—Ag40—S10	108.25 (13)
C67—Ag18—S18	122.2 (7)	O39—Ag41—S1	103.2 (2)
O35—Ag18—S17	97.3 (12)	O43—Ag41—O39	112.2 (5)
O35—Ag18—S18	131.1 (13)	O43—Ag41—S1	136.5 (4)
S17—Ag18—S18	101.82 (14)	O4—Ag42—O7	88.1 (3)
S13—Ag19—S14	141.55 (17)	O4—Ag42—S4	153.0 (2)
S13—Ag19—S15	105.91 (16)	O7—Ag42—S4	118.6 (2)
Symmetry code: (i) −*x*+1, −*y*+1, −*z*+1.			
**Ag84b**			
Ag1—Ag42	3.207 (12)	Ag20—S22^{i}	2.796 (5)
Ag1—S1	2.33 (3)	Ag20—O41	2.27 (2)
Ag1—S22^{i}	2.656 (14)	Ag21—Ag22	3.003 (2)
Ag1—O4	2.578 (16)	Ag21—S2	2.407 (8)
Ag2—Ag3	2.853 (5)	Ag21—S3	2.437 (7)

Ag2—S2	2.530 (9)	Ag22—Ag23	3.181 (3)
Ag2—S11	2.428 (7)	Ag22—S3	2.421 (5)
Ag2—O6	2.412 (11)	Ag22—O21	2.156 (10)
Ag3—Ag21	3.317 (5)	Ag23—S6	2.406 (6)
Ag3—Ag25	3.348 (4)	Ag23—S10	2.416 (7)
Ag3—Ag30	3.270 (6)	Ag24—S8	2.675 (8)
Ag3—S2	2.152 (10)	Ag24—S13	2.537 (7)
Ag3—O26	2.310 (11)	Ag24—O23	2.475 (10)
Ag3—O29	2.49 (3)	Ag24—O27	2.26 (2)
Ag4—Ag23	3.183 (5)	Ag25—Ag26	3.353 (3)
Ag4—Ag24	2.993 (3)	Ag25—Ag30	2.999 (3)
Ag4—Ag25	2.723 (4)	Ag25—S10	2.516 (9)
Ag4—Ag26	2.998 (4)	Ag25—S12	2.421 (10)
Ag4—S10	2.666 (12)	Ag25—O26	2.490 (11)
Ag4—S13	2.255 (11)	Ag25—O30	2.53 (3)
Ag5—Ag29	2.618 (6)	Ag26—S12	2.624 (7)
Ag5—Ag32	3.304 (7)	Ag26—S13	2.490 (6)
Ag5—S16	2.466 (9)	Ag26—S16	2.744 (10)
Ag5—S17	2.336 (10)	Ag26—O25	2.493 (10)
Ag6—Ag7	3.064 (6)	Ag27—Ag28	2.959 (2)
Ag6—Ag24	2.876 (6)	Ag27—Ag29	3.095 (3)
Ag6—S8	2.628 (11)	Ag27—S15	2.516 (5)
Ag6—S13	2.247 (10)	Ag27—S16	2.423 (7)
Ag6—O19	2.490 (10)	Ag27—O24	2.530 (13)
Ag6—O31	2.53 (5)	Ag28—Ag29	2.901 (2)
Ag7—Ag15	3.276 (4)	Ag28—Ag34	3.1378 (19)
Ag7—Ag16	3.055 (5)	Ag28—S15	2.452 (5)
Ag7—S8	2.271 (9)	Ag28—S21	2.540 (5)
Ag7—S14	2.550 (9)	Ag28—O13	2.467 (12)
Ag8—Ag9	3.074 (3)	Ag28—O14	2.473 (12)
Ag8—Ag20^{i}	2.969 (2)	Ag29—Ag32	3.001 (3)
Ag8—S5^{i}	2.890 (6)	Ag29—S16	2.465 (8)
Ag8—S14	2.472 (7)	Ag29—S21	2.548 (5)
Ag8—S22	2.559 (4)	Ag29—O33	2.29 (2)
Ag8—O42^{i}	2.30 (2)	Ag30—Ag31	2.901 (3)
Ag9—Ag10	3.0078 (17)	Ag30—S11	2.389 (7)
Ag9—Ag13	3.0317 (19)	Ag30—S12	2.430 (8)
Ag9—Ag42^{i}	2.894 (3)	Ag31—S11	2.434 (7)
Ag9—S22	2.397 (5)	Ag31—S17	2.414 (7)
Ag9—O18	2.387 (14)	Ag31—S18	2.646 (6)
Ag9—O22	2.266 (12)	Ag32—Ag33	2.924 (3)
Ag10—Ag11	3.0424 (17)	Ag32—S17	2.439 (8)
Ag10—Ag12^{i}	2.7466 (18)	Ag32—S21	2.949 (6)

Ag10—Ag12	2.7410 (15)	Ag32—O34	2.29 (2)
Ag10—Ag13	2.9128 (17)	Ag32—O44	2.349 (18)
Ag10—Ag13[i]	2.8226 (17)	Ag33—Ag34	3.119 (2)
Ag10—O8	2.324 (10)	Ag33—Ag39	2.933 (2)
Ag10—O17	2.311 (9)	Ag33—S18	2.471 (7)
Ag11—Ag12	3.2691 (18)	Ag33—S21	2.446 (6)
Ag11—Ag13	2.961 (2)	Ag33—O43	2.322 (18)
Ag11—Ag19[i]	3.344 (3)	Ag34—Ag35	2.919 (2)
Ag11—Ag20[i]	3.1800 (19)	Ag34—Ag39	3.041 (2)
Ag11—Ag22[i]	3.147 (2)	Ag34—S20	2.733 (5)
Ag11—S22	2.374 (4)	Ag34—S21	2.614 (5)
Ag11—O3	2.147 (10)	Ag34—O13	2.391 (12)
Ag11—O9	2.469 (12)	Ag34—O35	2.349 (16)
Ag12—Ag13	2.7562 (16)	Ag35—Ag36	2.850 (2)
Ag12—Ag13[i]	2.7727 (17)	Ag35—S4[i]	2.650 (5)
Ag12—Ag42	3.3603 (18)	Ag35—S20	2.484 (5)
Ag12—O5	2.327 (11)	Ag35—O36	2.253 (16)
Ag12—O21[i]	2.302 (9)	Ag36—Ag37	2.966 (2)
Ag13—S22	2.552 (4)	Ag36—S4[i]	2.478 (5)
Ag13—O4[i]	2.406 (12)	Ag36—O1	2.392 (15)
Ag13—O16[i]	2.543 (11)	Ag36—O38	2.191 (14)
Ag14—Ag15	2.946 (2)	Ag37—Ag39	3.325 (2)
Ag14—S5[i]	2.448 (5)	Ag37—Ag41	3.169 (3)
Ag14—S15	2.466 (5)	Ag37—S19	2.504 (6)
Ag14—O9	2.548 (10)	Ag37—S20	2.561 (6)
Ag15—S14	2.450 (7)	Ag37—O37	2.280 (15)
Ag15—S15	2.550 (5)	Ag38—Ag39	2.954 (3)
Ag15—O32	2.39 (5)	Ag38—Ag40	2.986 (4)
Ag16—Ag17	3.259 (3)	Ag38—S18	2.479 (7)
Ag16—S7	2.349 (9)	Ag38—S19	2.567 (7)
Ag16—S8	2.377 (10)	Ag38—O6	2.414 (11)
Ag17—S6	2.395 (6)	Ag38—O39	2.37 (2)
Ag17—S7	2.396 (6)	Ag39—S18	2.460 (6)
Ag18—Ag22	3.037 (2)	Ag39—S20	2.426 (5)
Ag18—S3	2.939 (6)	Ag39—O11	2.524 (11)
Ag18—S4	2.433 (6)	Ag40—Ag41	2.979 (3)
Ag18—S6	2.423 (5)	Ag40—S1	2.29 (3)
Ag18—O3[i]	2.497 (12)	Ag40—S19	2.611 (8)
Ag19—Ag20	3.165 (3)	Ag40—O40	2.31 (3)
Ag19—Ag35[i]	3.212 (2)	Ag41—Ag42	3.213 (4)
Ag19—S4	2.388 (5)	Ag41—S1	2.94 (3)
Ag19—S5	2.386 (5)	Ag41—S7[i]	2.419 (7)
Ag20—Ag8[i]	2.969 (2)	Ag41—S19	2.406 (8)

Ag20—Ag21	3.180 (3)	Ag42—S1	2.36 (3)
Ag20—S3	2.513 (5)	Ag42—O5	2.238 (12)
Ag20—S5	2.542 (6)	Ag42—O22^{i}	2.414 (12)
S1—Ag1—S22^{i}	128.3 (10)	S12—Ag25—O26	102.6 (4)
S1—Ag1—O4	85.0 (9)	S12—Ag25—O30	94.0 (7)
O4—Ag1—S22^{i}	91.5 (5)	O26—Ag25—S10	87.7 (4)
S11—Ag2—S2	136.0 (3)	O26—Ag25—O30	100.2 (7)
O6—Ag2—S2	106.2 (3)	S12—Ag26—S16	91.2 (3)
O6—Ag2—S11	110.6 (4)	S13—Ag26—S12	124.7 (3)
S2—Ag3—O26	105.1 (4)	S13—Ag26—S16	113.0 (3)
S2—Ag3—O29	121.0 (8)	S13—Ag26—O25	116.9 (3)
O26—Ag3—O29	125.7 (7)	O25—Ag26—S12	95.9 (3)
S13—Ag4—S10	173.6 (3)	O25—Ag26—S16	111.8 (3)
S17—Ag5—S16	157.1 (4)	S15—Ag27—O24	77.0 (3)
S13—Ag6—S8	115.7 (4)	S16—Ag27—S15	145.16 (18)
S13—Ag6—O19	85.3 (3)	S16—Ag27—O24	88.7 (4)
S13—Ag6—O31	112.3 (12)	S15—Ag28—S21	144.9 (2)
O19—Ag6—S8	88.5 (3)	S15—Ag28—O13	102.1 (3)
O19—Ag6—O31	129.3 (13)	S15—Ag28—O14	124.9 (3)
O31—Ag6—S8	120.2 (13)	O13—Ag28—S21	99.3 (3)
S8—Ag7—S14	148.2 (3)	O13—Ag28—O14	79.6 (4)
S14—Ag8—S5^{i}	103.90 (19)	O14—Ag28—S21	86.2 (3)
S14—Ag8—S22	132.6 (2)	S16—Ag29—S21	141.6 (2)
S22—Ag8—S5^{i}	95.70 (14)	O33—Ag29—S16	115.0 (6)
O42^{i}—Ag8—S5^{i}	103.8 (7)	O33—Ag29—S21	102.7 (6)
O42^{i}—Ag8—S14	114.1 (6)	S11—Ag30—S12	158.3 (3)
O42^{i}—Ag8—S22	102.1 (6)	S11—Ag31—S18	105.1 (2)
O18—Ag9—S22	119.4 (3)	S17—Ag31—S11	153.6 (2)
O22—Ag9—S22	153.5 (4)	S17—Ag31—S18	101.2 (2)
O22—Ag9—O18	86.5 (4)	S17—Ag32—S21	110.6 (2)
O17—Ag10—O8	104.0 (4)	O34—Ag32—S17	129.0 (6)
S22—Ag11—O9	112.1 (3)	O34—Ag32—S21	101.3 (6)
O3—Ag11—S22	164.3 (3)	O34—Ag32—O44	93.6 (9)
O3—Ag11—O9	82.3 (4)	O44—Ag32—S17	113.4 (6)
O21^{i}—Ag12—O5	77.8 (4)	O44—Ag32—S21	106.0 (5)
O4^{i}—Ag13—S22	98.3 (3)	S21—Ag33—S18	148.9 (2)
O4^{i}—Ag13—O16^{i}	77.7 (4)	O43—Ag33—S18	100.2 (6)
O16^{i}—Ag13—S22	95.7 (3)	O43—Ag33—S21	110.9 (6)
S5^{i}—Ag14—S15	141.32 (17)	S21—Ag34—S20	145.71 (17)
S5^{i}—Ag14—O9	80.1 (2)	O13—Ag34—S20	91.2 (3)
S15—Ag14—O9	129.0 (3)	O13—Ag34—S21	99.3 (3)
S14—Ag15—S15	134.0 (2)	O35—Ag34—S20	109.5 (4)
O32—Ag15—S14	109.8 (14)	O35—Ag34—S21	99.9 (4)

O32—Ag15—S15	103.8 (16)	O35—Ag34—O13	103.0 (5)
S7—Ag16—S8	167.1 (3)	S20—Ag35—S4^{i}	123.43 (16)
S6—Ag17—S7	155.92 (19)	O36—Ag35—S4^{i}	91.0 (5)
S4—Ag18—S3	109.82 (16)	O36—Ag35—S20	138.9 (5)
S4—Ag18—O3^{i}	89.6 (4)	O1—Ag36—S4^{i}	104.5 (3)
S6—Ag18—S3	100.73 (18)	O38—Ag36—S4^{i}	130.1 (5)
S6—Ag18—S4	145.79 (18)	O38—Ag36—O1	110.7 (6)
S6—Ag18—O3^{i}	107.1 (3)	S19—Ag37—S20	126.5 (2)
O3^{i}—Ag18—S3	87.5 (3)	O37—Ag37—S19	107.8 (5)
S5—Ag19—S4	164.07 (18)	O37—Ag37—S20	116.0 (5)
S3—Ag20—S5	133.4 (2)	S18—Ag38—S19	129.7 (2)
S3—Ag20—S22^{i}	93.67 (16)	O6—Ag38—S18	114.7 (3)
S5—Ag20—S22^{i}	98.46 (15)	O6—Ag38—S19	95.5 (3)
O41—Ag20—S3	111.6 (6)	O39—Ag38—S18	110.7 (7)
O41—Ag20—S5	106.3 (6)	O39—Ag38—S19	92.1 (6)
O41—Ag20—S22^{i}	109.4 (7)	O39—Ag38—O6	111.8 (7)
S2—Ag21—S3	158.9 (2)	S18—Ag39—O11	102.5 (3)
O21—Ag22—S3	169.5 (3)	S20—Ag39—S18	143.4 (2)
S6—Ag23—S10	151.5 (4)	S20—Ag39—O11	113.9 (3)
S13—Ag24—S8	104.9 (3)	S1—Ag40—S19	116.7 (7)
O23—Ag24—S8	81.0 (3)	S1—Ag40—O40	125.3 (10)
O23—Ag24—S13	104.9 (3)	O40—Ag40—S19	115.9 (7)
O27—Ag24—S8	108.5 (6)	S7^{i}—Ag41—S1	94.9 (7)
O27—Ag24—S13	141.0 (6)	S19—Ag41—S1	102.3 (7)
O27—Ag24—O23	99.8 (7)	S19—Ag41—S7^{i}	154.2 (2)
S10—Ag25—O30	96.8 (7)	O5—Ag42—S1	122.9 (8)
S12—Ag25—S10	163.5 (3)	O5—Ag42—O22^{i}	94.0 (4)
Symmetry code: (i) $-x+2$, $-y$, $-z$.			

Reference:

1 Z. Wang, H.-F. Su, Y.-Z. Tan,; S. Schein, S.-C. Lin, W. Liu, S.-A. Wang, W.-G. Wang, C.-H. Tung, D. Sun, and L.-S. Zheng, *Proc Natl Acad Sci USA* 2017, **114**, 12132-12137.

2 L. Palatinus and G. Chapuis, *J. Appl. Crystallogr.* 2007, **40**, 786-790.

3 G. M. Sheldrick, *Acta. Crystallogr., Sect. C* 2015, **71**, 3-8.

4 O. V. Dolomanov, L. J. Bourhis, R. J. Gildea, J. A. K. Howard, and H. Puschmann, *J. Appl. Crystallogr.* 2009, **42**, 339-341.

5 A. L. Spek, *Acta. Crystallogr., Sect. D*. 2009, **65**, 148-155.

6 B. Delley, *J. Chem. Phys.,* 1990, **92**, 508-517.

7 B. Delley, *J. Chem. Phys.,* 2000, **113**, 7756-7764.

8 P. E. Blohl, *Phys. Rev. B,* 1994, **50**, 17953-11979.

9 B. Delley, *Chem. Phys.,* 1986, **110**, 329-338.

10 G. Kresse and J. Furthmüller, *Phys. Rev. B*, 1996, **54**, 11169-11186.

11 J. P. Perdew, K. Burke and M. Ernzerhof, *Phys. Rev. Lett.,* 1996, **77**, 3865-3868.
